# Interleukin 21 collaborates with interferon-γ for the optimal expression of interferon-stimulated genes and enhances protection against enteric microbial infection

**DOI:** 10.1371/journal.ppat.1007614

**Published:** 2019-02-28

**Authors:** Shahram Solaymani-Mohammadi, Jay A. Berzofsky

**Affiliations:** Vaccine Branch, Center for Cancer Research, National Cancer Institute, National Institutes of Health, Bethesda, MD, United States of America; University of Utah, UNITED STATES

## Abstract

The mucosal surface of the intestinal tract represents a major entry route for many microbes. Despite recent progress in the understanding of the IL-21/IL-21R signaling axis in the generation of germinal center B cells, the roles played by this signaling pathway in the context of enteric microbial infections is not well-understood. Here, we demonstrate that *Il21r*^-/-^ mice are more susceptible to colonic microbial infection, and in the process discovered that the IL-21/IL-21R signaling axis surprisingly collaborates with the IFN-γ/IFN-γR signaling pathway to enhance the expression of interferon-stimulated genes (ISGs) required for protection, via amplifying activation of STAT1 in mucosal CD4^+^ T cells in a murine model of *Citrobacter rodentium* colitis. As expected, conditional deletion of STAT3 in CD4^+^ T cells indicated that STAT3 also contributed importantly to host defense against *C*. *rodentium* infection in the colon. However, the collaboration between IL-21 and IFN-γ to enhance the phosphorylation of STAT1 and upregulate ISGs was independent of STAT3. Unveiling this previously unreported crosstalk between these two cytokine networks and their downstream genes induced will provide insight into the development of novel therapeutic targets for colonic infections, inflammatory bowel disease, and promotion of mucosal vaccine efficacy.

## Author summary

Diarrheal diseases still remain the second leading cause of mortality in children younger than 5 years old worldwide, leading to 1.3 million deaths per annum. The diarrheagenic *Escherichia coli* (DEC) pathotypes are considered NIAID Biodefense Category B agents. Human infections with enteropathogenic and enterohemorrhagic *Escherichia coli* (EPEC and EHEC, respectively) are associated with human disease. EPEC is a common cause of infantile diarrhea in the developing and underdeveloped world, and EHEC is considered an emerging zoonotic infection. These enteric pathogens cause a wide range of clinical symptoms, varying from mild diarrhea to more complicated clinical presentations, including hemolytic-uremic syndrome (HUS) and hemorrhagic colitis. Using a murine model of *Citrobacter rodentium* infection, we found the requirement of a functional IL-21/IL-21R signaling axis in the control of enteric microbial infections via augmenting activation of STAT1 in mucosal CD4^+^ T cells in a murine model of *Citrobacter rodentium* colitis. Understanding how the IL-21/IL-21R signaling pathway contributes to the host immunity in the colon will further provide insights into the development of novel preventive and therapeutic targets for human subjects with enteric microbial infections and other inflammatory conditions, including inflammatory bowel disease (IBD) and celiac disease.

## Introduction

Several microbial pathogens elicit the type I (e.g. IFN-α, IFN-β), type II (IFN-γ) or type III (e.g. IFN-λ) interferons, leading to the transcription of several hundred interferon-stimulated genes (ISGs) [1, 2]. The activation of each of these interferon systems induces a distinct but partially overlapping set of signature ISGs which have been reported to contribute important roles to host defense against a wide variety of pathogens, including viruses, bacteria, and parasites, by directly targeting genes conferring resistance to infection [3, 4]. The essential requirement of ISGs in host immunity has been shown by the fact that mice deficient in one or more ISGs are more susceptible to infections with multiple viral and bacterial pathogens [5–7]. The role of ISGs in host defense at mucosal surfaces of the gut is not fully understood. In an attempt to elucidate the mechanisms by which infection is controlled at mucosal surfaces of the small intestine, the cooperation between two other cytokine networks, interferon lambda (IFN-λ) (not IFN-γ) and IL-22, was shown to be required for optimal control of viral replication in a mouse model of rotavirus infection [6]. However, the role of the IL-21/IL-21R signaling axis in colitis of any origin and in collaboration with IFN-γ is not known.

Infection of mice with the murine enteric pathogen, *Citrobacter rodentium*, is considered a robust model to study host immune response to minimally-invasive gut pathogens at the mucosal surfaces of the large intestine [8–10]. This extracellular bacterial pathogen shares virulence features with the closely related human enteropathogenic *Escherichia coli* (EPEC) and enterohemorrhagic *Escherichia coli* (EHEC), making it an ideal surrogate model to study the human disease [8, 10]. Several lines of evidence suggest that the immune response to the human EPEC and EHEC relies on both innate [11–13] and adaptive [14–17] arms of the immune system. The *C*. *rodentium* colonization elicits a robust T_H1_ response, characterized by the predominant production of IFN-γ, IL-12 [14] and TNF-α [18], as well as a T_H17_ response producing IL-17 [19]. The absolute requirement of T_H1_ responses in host defense against *C*. *rodentium* has been reinforced by the observations that CD4^+^ T cell-deficient mice (CD4^-/-^) (but not CD8^+^ T cell-deficient mice) IFN-γ knockout (*Ifng*^-/-^) mice and IL-12-deficient mice (IL-12p40^-/-^) showed greater susceptibility to enteric infection, increased systemic dissemination of the bacterium and enhanced pathology after *C*. *rodentium* infection [16, 17, 20].

IL-21 binding to its cognate receptor, IL-21R, results in the activation of Janus kinase 1 (JAK1) and JAK3 and the subsequent phosphorylation of signal transducer and activator of transcription (STAT) proteins, mainly STAT3 but also STAT1 and STAT5 [21, 22]. The activated phospho-STAT proteins dimerize and translocate into the nucleus, bind to the interferon (IFN)-γ-activated sequence (GAS) motif and initiate a transcription program that includes some IL-21 target genes [23]. Although the cascade of events occurring after the IL-21-activation of STAT3 is well-studied, it is still not fully understood how the activation of STAT1 via IL-21 influences downstream target genes [23]. However, it is interesting that IL-21 via STAT1 can augment expression of both *Tbx21* and *Ifng* gene expression as well as expression of certain interferon-regulated genes *Ifit1* and *Ifit2*, that are also IL-21 targets, and that STAT3 activation diminishes these effects, either in mice or humans [24].

Despite the critical requirement of the IL-21/IL-21R signaling axis in the generation of germinal center B cells [25], the roles played by this signaling pathway in the context of enteric microbial infection is not well-characterized. Based on this, we investigated the role of IL-21/IL-21R axis, in collaboration with the type II interferon, IFN-γ, in protection against enteric microbial infection with *C*. *rodentium* in the colon. We found that an intact IL-21/IL-21R axis was required for resistance against and clearance of enteric infection with this pathogen and that activated CD4^+^ T cells were the exclusive expressors of IL-21 following infection with *C*. *rodentium* in the distal colon. The CD4^+^ T cell-derived IL-21 curtailed enteric infection and also contributed to *C*. *rodentium*-induced inflammation and pathology at the mucosal surfaces of the colon. We also found that IL-21 acted in concert with IFN-γ to optimally activate STAT1 in CD4^+^ T cells and to promote subsequent optimal expression of ISGs in the distal colon. These events were independent of STAT3. Our findings revealed a previously unknown effector function for the IL-21/IL-21R signaling axis in amplification of ISG expression and the modulation of host response to microbial infection at the mucosal surfaces of the gut. The understanding of the mechanisms by which the IL-21/IL-21R signaling axis regulates intestinal epithelial integrity and host immunity after infection with minimally-invasive gut pathogens (e.g. *Escherichia coli*) will provide insights into novel preventive and therapeutic targets for the control of human infections with enteric bacterial pathogens. Such collaboration between IL-21 and IFN-γ also provides mechanisms by which the IL-21/IL-21R signaling axis regulates inflammation in the colon and provides insights into novel preventive and therapeutic targets for inflammatory conditions in humans, including inflammatory bowel disease, as well as inflammation-induced cancers.

## Results

### An intact IL-21/IL-21R signaling axis is required for optimal host protection against colonic *Citrobacter rodentium* infection

**Fig 1:**
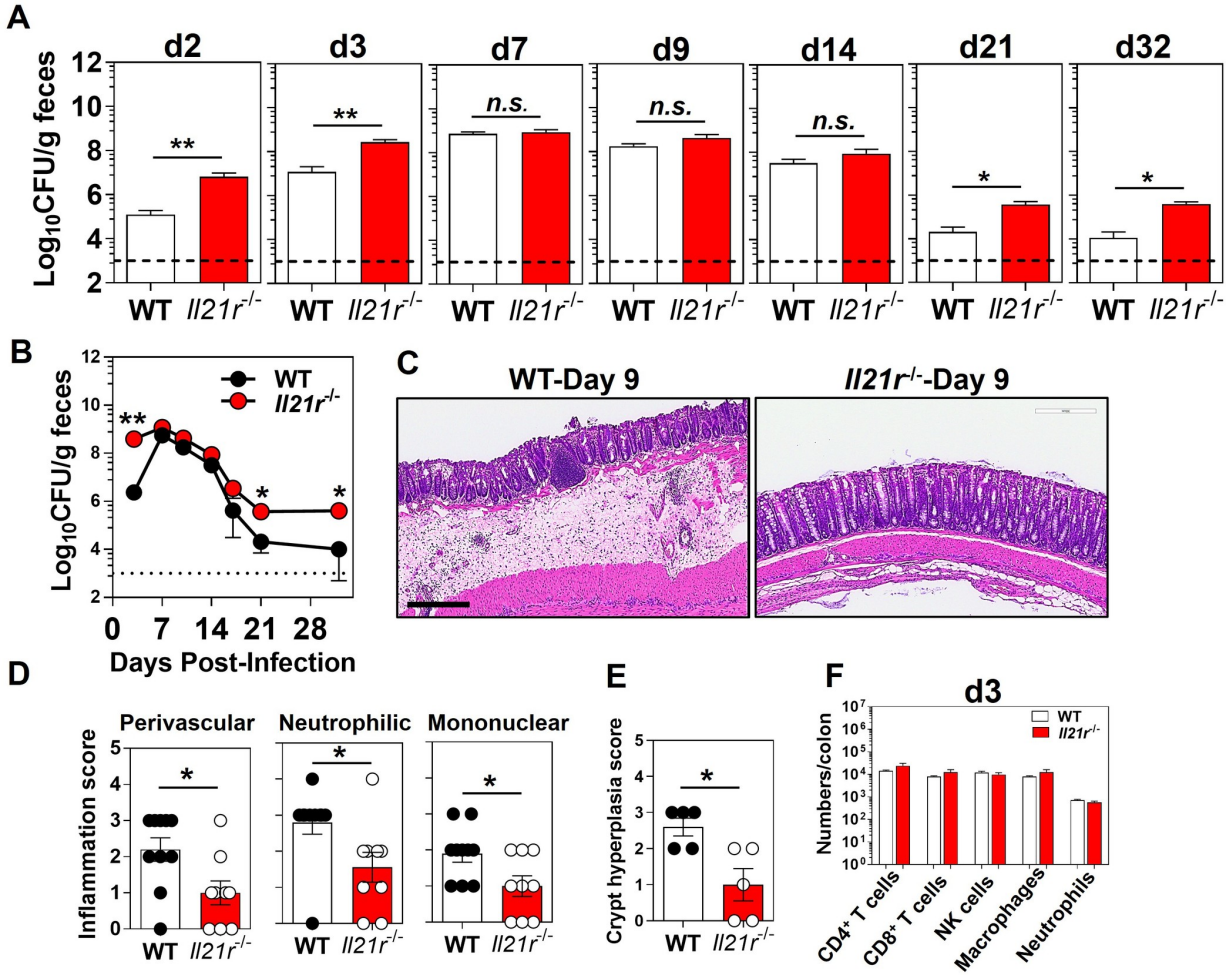
An intact IL-21/IL-21R signaling axis is required for host protection against colon *Citrobacter rodentium* infection. **A**. Bacterial burden and **B**. infection kinetics in the feces of *Il21r*^-/-^ mice and WT controls shown as colony forming unit (CFU)/g feces. The results are the Mean ± SEM of 5–13 mice per group pooled from two of four independent experiments with similar results. The dashed line represents the sensitivity of the culture method. *p < 0.05; **p < 0.001 determined by Mann-Whitney *U* test. **C**. Representative colonic pathology as assessed by hematoxylin and eosin (H&E) staining (5×). The scale bar, 300 μm. **D**. Inflammation scoring of distal colon of *Il21r*^-/-^ mice and WT controls at 9 days after infection with *C*. *rodentium*. Data are the Mean ± SEM from two pooled independent experiments with a total of 9 (*Il21r*^-/-^) or 10 (WT) mice/group. *p < 0.05 determined by Mann-Whitney *U* test. **E**. Histological scoring of crypt hyperplasia in distal colon of *Il21r*^-/-^ mice and WT controls at 9 days after infection with *C*. *rodentium*. Data are representative of one of two independent experiments with a total of 5 (*Il21r*^-/-^) or 5 (WT) mice/group. *p < 0.05 determined by Mann-Whitney *U* test. **F**. The absolute numbers of immune subsets in the whole colon LP of *Il21r*^-/-^ mice and WT controls at 3 days p.i. Data are the Mean ± SEM of absolute numbers of immune cell types, analyzed by Mann-Whitney *U* test.

Using the murine intestinal pathogen, *C*. *rodentium*, we investigated the requirement of the IL-21/IL-21R signaling axis in protection against mucosal microbial infection in the gut. Our findings indicated that *Il21r*^-/-^ mice had significantly higher (2 logs) bacterial burden in the feces as compared with WT controls, both early and late in the infection, although the peak bacterial load was comparable (Fig 1A and 1B). While the WT controls were able to control the infection with *C*. *rodentium* by day 21 p.i., *Il21r*^-/-^ mice had an impaired ability to clear the enteric infection with this pathogen (Fig 1A and 1B). The differences in fecal bacterial burdens were noticeable as early as day 2 p.i. in *Il21r*^-/-^mice, suggesting an important roleplayed by this signaling pathway in early host protection events to this enteric pathogen. Although the WT and *Il21r*^-/-^ mice were cohoused for 2 weeks to equilibrate microbiota, we also bred matched WT heterozygotes and homozygous *Il21r*^-/-^ mice from the same set of homozygous *Il21r*^-/-^ females by crossing with WT or *Il21r*^-/-^ males, and then kept the litters nursing together until weaned, so that they would obtain the same microbiota from their mothers intrapartum and during nursing/foster nursing. Consistently, mice homozygous for the targeted mutation of IL-21R (*Il21r*^-/-^) showed significantly higher bacterial burdens and delayed clearance of *C*. *rodentium* infection as compared with their heterozygous littermate controls (*Il21*^-/+^) that were bred together (S1 Fig), confirming that the difference in susceptibility to *C*. *rodentium* infection was not due to distinct microbiomes. Furthermore, comparable infection kinetics were observed following infection with an OVA-expressing *C*. *rodentium*, indicating that overexpression of a plasmid carrying the chicken ovalbumin did not alter the infectivity, or the ability to colonize the mucosal surfaces, of OVA-*Citrobacter* as compared with WT-*Citrobacter* (S2 Fig). Collectively, these findings were consistent with one previous report indicating a role played by the IL-21/IL-21R axis in protection against *C*. *rodentium* [26].

Despite effective bacterial replication and significantly higher bacterial burden in *Il21r*^-/-^ mice, histology demonstrated only a moderate increase in inflammatory cell recruitment, predominantly in the mucosa, and mild hyperplasia accompanied with loss of goblet cells in the distal colons of *Il21r*^-/-^ mice 9 days after infection with *C*. *rodentium*, the time of peak infection when bacterial loads are not significantly different between WT and *Il21r*^-/-^ mice (Fig 1A–1C). In WT controls increased numbers of inflammatory cells were observed in the mucosa extending into the submucosa of the distal colon. Epithelial cell erosion and ulcerations, and submucosa edema, were more prominent in WT mice than in their *Il21r*^-/-^ counterparts at day 9 p.i. (Fig 1C). Crypt hyperplasia and loss of goblet cells accompanied by significant submucosal edema with significantly higher perivascular inflammatory cells were more noticeable in the distal colon of WT mice 9 days after infection than in the *Il21r*^-/-^ mice (Fig 1D and 1E). However, the percentage and the absolute numbers of those cells were comparable in whole colons between the two genotypes at days 3 and 9 after *C*. *rodentium* infection (Fig 1F and S3A–S3G Fig). Overall, despite having higher bacterial burden, the *Il21r*^-/-^ mice appeared to have less inflammation, indicating that IL-21 response was a critical factor in the inflammatory response and at least part of that inflammatory response may be necessary for control of the bacterial load.

### IL-21 is induced in distal colon following infection with *C*. *rodentium*

**Fig 2:**
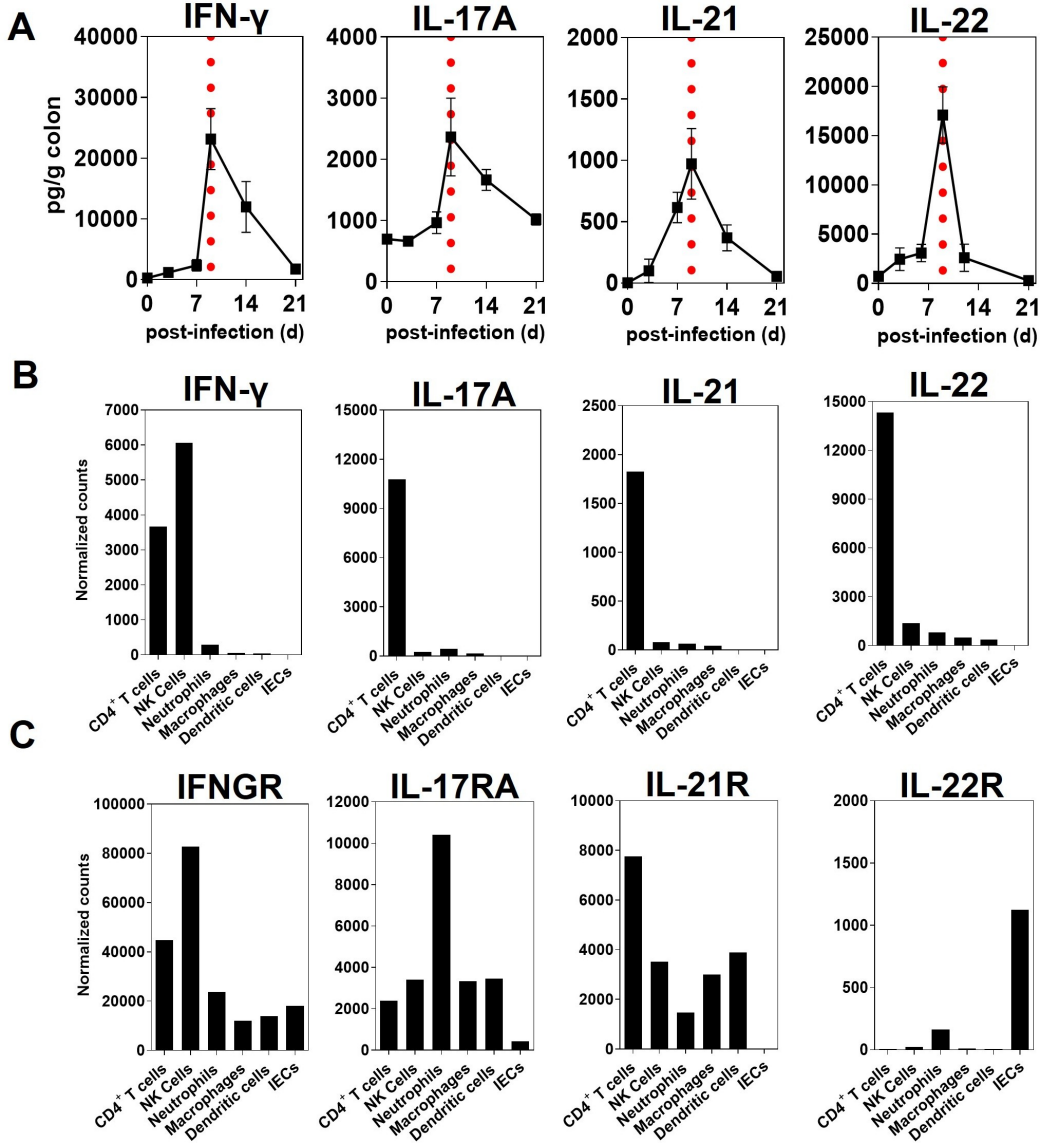
IL-21 is induced in the distal colon following infection with *C*. *rodentium*. **A**. Cytokine ELISA of the indicated cytokines from supernatants of the *ex-vivo* organ culture of the colonic tissues from WT mice collected at different time-points p.i. (48 hr cultures). Data are Mean ± SEM from two or more experiments (*n* = 7 mice/time-point). Day 9 after infection is indicated by vertical dashed lines. **B**. The mRNA expression of indicated cytokines (IFN-γ, IL-17A, IL-21, and IL-22) and **C**. Cytokine receptors (IFN-γR, IL-17RA, IL-21R, and IL-22R) by immune cells in the LP and non-immune epithelial cells (IECs) in the pooled distal colon of WT mice (*n* = 25) 9 days after infection with *C*. *rodentium* analyzed by Nanostring.

Using an *ex-vivo* organ culture system, we examined the kinetics of IL-21 production by ELISA in the distal colon of WT mice following *C*. *rodentium* infection. As shown in Fig 2A, the peak of IL-21 production in the distal colon of WT mice infected with *C*. *rodentium* occurred 9 days p.i., when the infection was at its peak. Likewise, similar kinetics were observed for other cytokines important for protection against *C*. *rodentium* infection, including IFN-γ, IL-17A and IL-22 in the distal colon of WT (Fig 2A). To further investigate the extent to which immune or non-immune cell types in the distal colon contributed to the expression of IL-21 after *C*. *rodentium* infection, we sorted cells in the colon into hematopoietic (CD45^+^EpCAM^-^) or non-hematopoietic (CD45^-^EpCAM^+^) cells and sorted for immune cell types. Using the Nanostring method, we observed that IL-21 was almost exclusively expressed by mucosal CD4^+^ T cells (CD45^+^EpCAM^-^CD3^+^CD4^+)^ in WT mice 9 days after *C*. *rodentium* infection, while other cells of hematopoietic origin, such as natural killer (NK) cells, dendritic cells (DCs), neutrophils, and macrophages, did not express significant levels of IL-21 transcripts following infection (Fig 2B). Furthermore, mucosal CD4^+^ T cells expressed higher levels of transcripts for IL-21R as compared with other immune and non-immune cells after infection with *C*. *rodentium* (Fig 2C**)**. However, there was some IL-21R expression by innate cells such as NK cells, neutrophils, macrophages and DCs that may contribute to the difference in early control of *C*. *rodentium* at day 2–3 pi. (Fig 1A and 1B). Interestingly, the colonic intestinal epithelial cells (IECs) expressed neither detectable transcripts for IL-21 (Fig 2B) nor mRNA for IL-21R following infection (Fig 2C). The latter observations were consistent with lack of IL-21R expression in a C57BL/6 colon carcinoma cell line (MC-38) model of colonic IECs (S4 Fig).

### IL-21/IL-21R signaling axis is critical for the optimal expression of ISGs following colon infection

**Fig 3:**
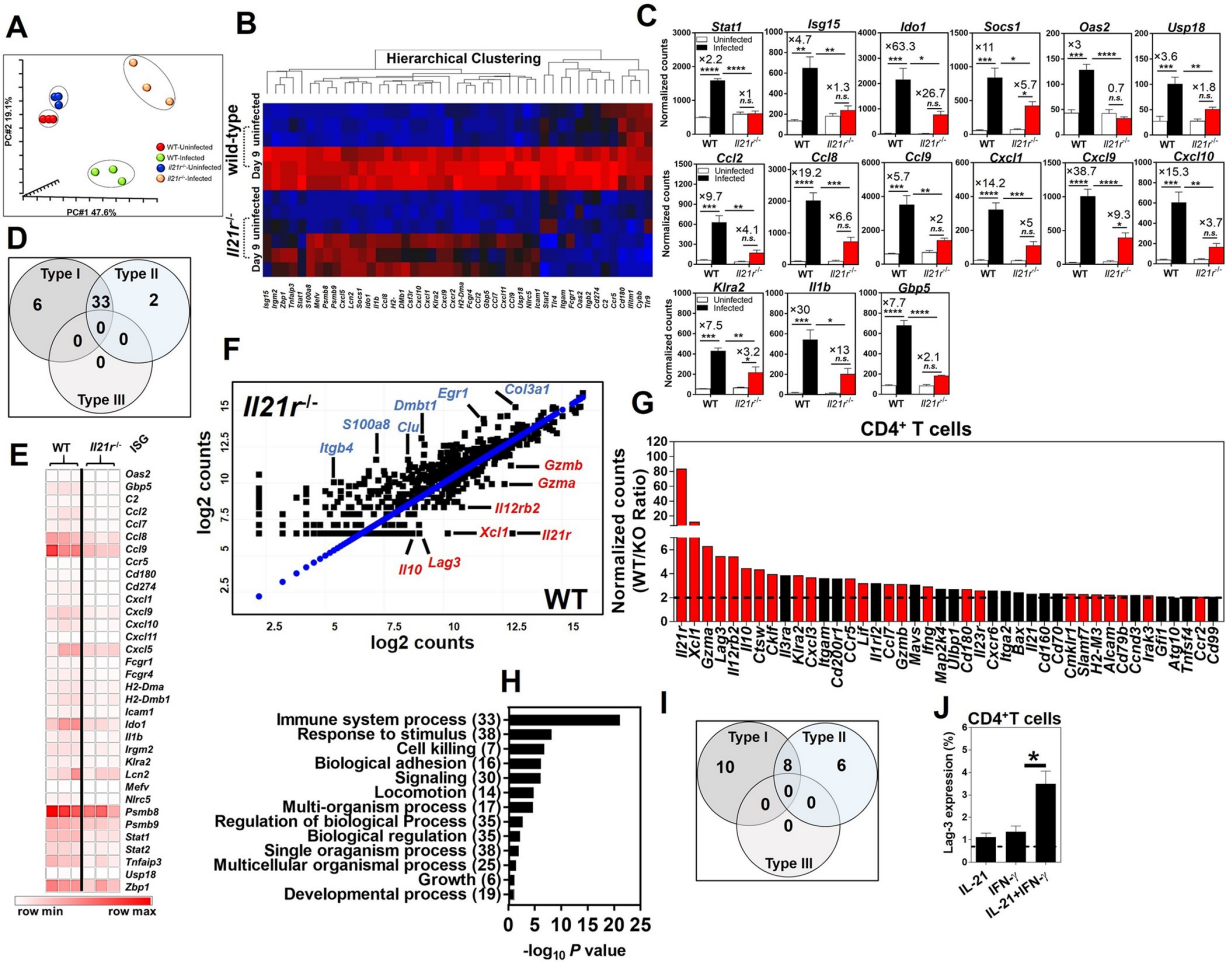
IL-21/IL-21R signaling axis is critical for the optimal expression of ISGs following colon infection. **A**. Principal component analysis (PCA) of Nanostring gene expression datasets from whole distal colons of uninfected and infected WT and *Il21r*^-/-^ mice at 9 days p.i. (*n* = 3/genotype/time-point). **B**. Heatmap of differential gene expression profiles in the whole distal colon of uninfected and infected WT and *Il21r*^-/-^ mice at 9 days p.i. (red, upregulated; blue, downregulated). **C**. Normalized expression levels of ISGs in the whole distal colon of uninfected versus infected WT and *Il21r*^-/-^ mice at 9 days p.i. (*n* = 3/genotype/time-point). The gene expression of each gene was normalized to the geometric mean of the expression of internal reference genes and presented as normalized counts/gene/biological sample (see Nanostring Methods). Genes with fold difference ≥ 2 between WT/*Il21r*^-/-^ mice were considered. Data represent the mean ± SEM of normalized counts. *p < 0.05; **p < 0.01; ***p < 0.001; ****p < 0.0001; One-way ANOVA followed by Bonferroni post-hoc adjustment test for multiple comparison. **D**. Venn diagram representing the number of type I-, type II- and type I/II-specific ISGs, as analyzed using the Interferome Database with impaired upregulation during infection in the whole distal colon of *Il21r*^-/-^ mice 9 days p.i. versus WT controls. **E**. Heatmap of differential expression of ISGs in the whole distal colon of *Il21r*^-/-^ mice as compared with WT controls at 9 days p.i. **F**. Scatter plot representation (log_2_ values) of gene expression levels in FACS-sorted mucosal CD4^+^ T cells isolated from the distal colon of WT and *Il21r*^-/-^ mice at 9 days p.i. **G**. Histogram shows fold impairment of genes in rank order in *Il21r*^-/-^ mice versus WT controls (Bars in red represent known ISGs, and appear to be the dominant class of gene impaired). Black bars represent impaired genes in CD4^+^ T cells from *Il21r*^-/-^ mice that are not known ISGs. Genes with fold difference ≥ 2 between WT/*Il21r*^-/-^ mice were considered. **H**. Gene ontology analysis of processes enriched in mucosal CD4^+^ T cells from the distal colons of WT controls versus *Il21r*^-/-^ mice (WT/*Il21r*^-/-^ ratio) 9 days p.i. Genes with fold difference ≥ 2 were considered. **I**. Venn diagram representing the number of type I-, type II-, type III- and type I/II-specific ISGs impaired in FACS-sorted CD4^+^ T cells isolated from the distal colon of *Il21r*^-/-^ mice 9 days p.i., as analyzed using the Interferome Database. **J**. The induction of a representative ISG, LAG-3, in CD4^+^ T cells isolated from naïve *Il21r*^-/-^ mice and WT controls. Cells were cultured in the presence of mIFN-γ (20 ng/ml) or mIL-21 (20 ng/ml) alone or in combination for 24 hr and the surface expression of LAG-3 was determined by flow cytometry. Data are the Mean ± SEM from two pooled independent experiments with a total of 6 (*Il21r*^-/-^) or 6 (WT) mice/group. *p < 0.05 determined by Mann-Whitney *U* test.

We performed principal component analysis (PCA) of gene expression profiles in the whole distal colon of WT and *Il21r*^-/-^ mice using Nanostring. Our analysis showed four distinct clusters between uninfected and infected WT and *Il21r*^-/-^ mice 9 days after infection with *C*. *rodentium* (Fig 3A). While the uninfected WT and *Il21r*^-/-^ mice clustered closed to each other, the infected WT and *Il21r*^-/-^ mice formed two distinct clusters that were separated far from each other, indicating differences in their gene expression profiles.

Several lines of evidence suggest that colonization with *C*. *rodentium* elicits a robust, highly polarized T_H1_ response in the colon, as shown by increased expression of IFN-γ, tumor necrosis factor (TNF)-α and IL-12 [14]. The critical requirement of IFN-γ during *C*. *rodentium* infection has been highlighted by the observations that IFN-γ knockout (*Ifnγ*^-/-^) mice had an impaired ability to clear infection [14, 17]. In this model, IFN-γ produced by antigen-experienced CD4^+^ T cells mediates the mucosal immune response to *C*. *rodentium* and its subsequent eradication [20]. Our findings demonstrated that type I- and type II-specific, but not type III-specific, ISGs were induced after *C*. *rodentium* infection in the distal colons of WT mice (Fig 3B, 3C and 3D and S5 Fig). Considering the key requirement of IFN-γ and downstream ISGs in host defense against *C*. *rodentium* infection, we next investigated the contribution of IL-21/IL-21R signaling axis to the optimal expression of ISGs in the colon of *Il21r*^-/-^ mice under homeostatic conditions as well as 9 days after enteric infection. Our findings demonstrated that most genes impaired in the whole distal colon of *Il21r*^-/-^ mice infected with *C*. *rodentium* were known ISGs (Fig 3C and 3D). In each case, the fold increase during infection compared to uninfected controls was substantially less in the *Il21r*^-/-^ mice than in the WT controls (Fig 3C), and in most cases the increase in *Il21r*^-/-^ mice was not significant, and the increase in the WT was significantly greater than the increase in *Il21r*^-/-^ mice. Interestingly, the expression of both the type I- and type II-specific ISGs were impaired in the whole colon of *Il21r*^-/-^ mice 9 days after *C*. *rodentium* infection (Fig 3D and 3E).

To further explore the extent of impaired expression of ISGs in the absence of an intact IL-21/IL-21R axis specifically in CD4^+^ T cells, FACS-sorted CD4^+^ T cells (the main producers of IL-21 and main expressors of IL-21R, Fig 2B and 2C) were isolated from the distal colon lamina propria (LP) of *Il21r*^-/-^ mice and WT controls 9 days after *C*. *rodentium* infection and analyzed by Nanostring. Consistently, our findings indicated that the majority of genes impaired in CD4^+^ T cells isolated from the LP of *Il21r*^-/-^ mice were known ISGs (Fig 3F and 3G**)**, indicated by red bars (Fig 3G). Gene ontology analysis of processes enriched in mucosal CD4^+^ T cells impaired from the distal colons of *Il21r*^-/-^ mice identified genes with a wide range of functions (Fig 3H). Likewise, the expression of type I- and type II-specific, but not type III-specific, ISGs by CD4^+^ T cells was impaired in *Il21r*^-/-^ mice as compared with WT controls (Fig 3I).

In view of these results, we hypothesized that IL-21 and IFN-γ produced in response to *C*. *rodentium* infection may act in concert for the optimal expression of ISGs in the colon and are required for the control of *C*. *rodentium* infection *in vivo*. To experimentally test the hypothesis, we treated naïve splenocytes with recombinant murine IL-21 or IFN-γ alone or in combination for 24 hr and the expression of representative ISGs LAG3 and granzyme A by CD4^+^ T cells was measured by flow cytometry. When cells were treated with a combination of recombinant murine IL-21 or IFN-γ the expression of representative ISGs by CD4^+^ T cells isolated from WT mice was significantly upregulated compared to cells treated with either IL-21 or IFN-γ alone (Fig 3J for LAG-3 and S6 Fig for granzyme A). Interestingly, almost all CD4^+^ T cells positive for IFN-γR were also positive for IL-21R as well (S7 Fig), so the same cells could respond to both cytokines, not a sum of some cells that could respond to IFN-γ and some to IL-21. To further address whether differences in the gene expression profiles between WT and *Il21r*^-/-^ mice did not merely reflect the severity of inflammation, we investigated the expression of a representative ISG, LAG-3, expressed by CD4^+^ T cells isolated from the colonic LP of naïve (uninfected) WT and *Il21r*^-/-^ mice. Remarkably, *Il21r*^-/-^ mice expressed significantly lower surface expression of LAG-3 (*p* = 0.001; *n* = 7 animals) even in the absence of colonic inflammation (S8 Fig). These findings indicate that the differences in the gene expression profiles between WT and *Il21r*^-/-^ mice are not likely a consequence of the inflammation severity. Collectively, these findings indicate previously unrecognized collaboration between IL-21 and the IFN-γ signaling pathway to optimally express ISGs and a requirement for an intact IL-21/IL-21R signaling axis for the optimal expression of ISGs by CD4^+^ T cells.

### IFN-γ, but not IFN-α/β, is required for the clearance of infection with *C*. *rodentium*

**Fig 4:**
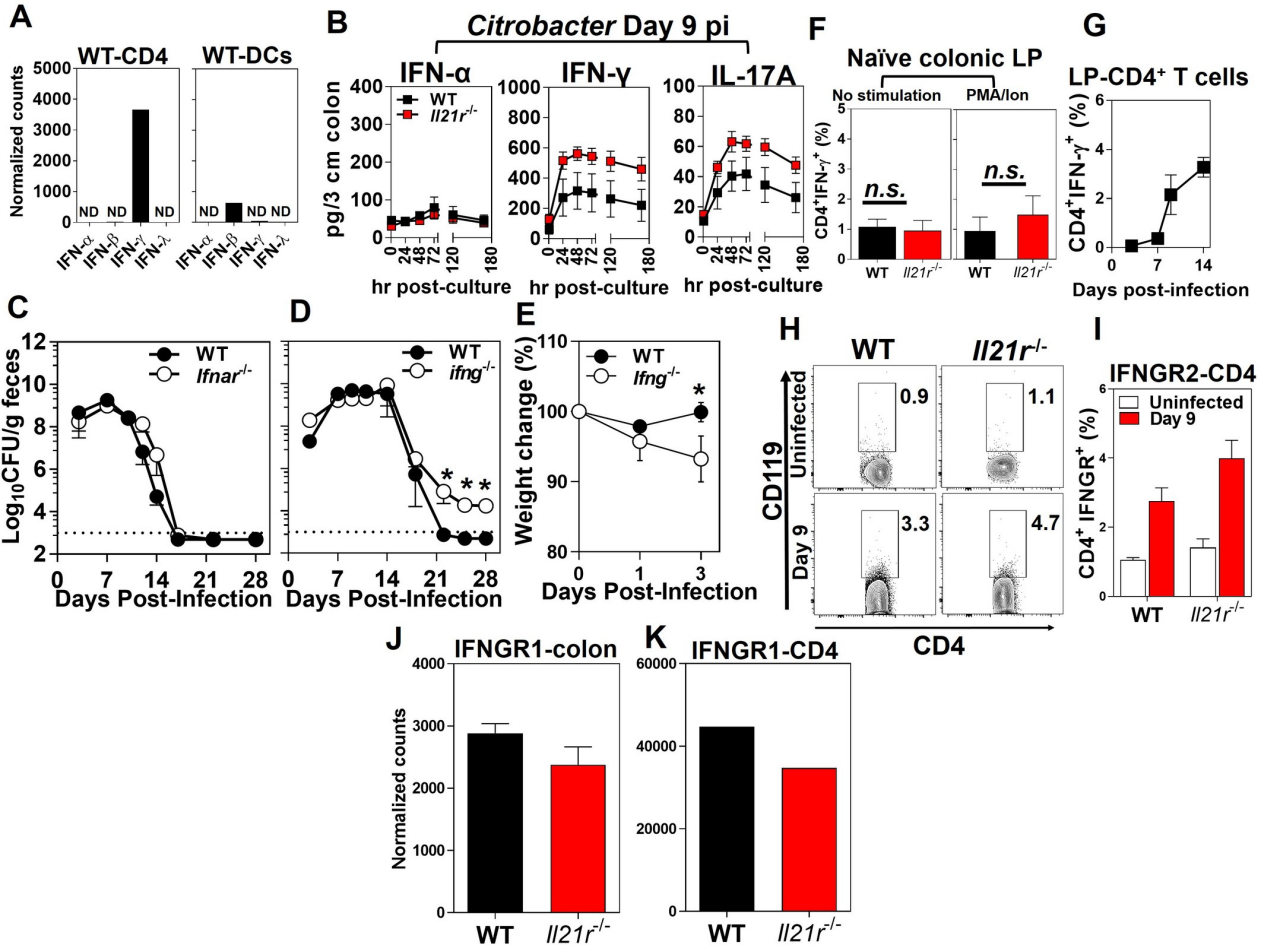
The requirement of IFN-γ, but not IFN-α/β, for the clearance of infection with *C*. *rodentium*. **A**. The expression levels of interferons (α, β, γ, λ) by the FACS-sorted CD4^+^ T cells (left) and DCs (right) isolated from the distal colonic LP of WT mice 9 days p.i. Results are from pooled distal colon LP of WT mice (*n* = 25) 9 days after infection with *C*. *rodentium* analyzed by Nanostring. N.D., not detected. **B**. Cytokine ELISA kinetics of IFN-α, IFN-γ and IL-17A from supernatants of the *ex-vivo* organ culture of the colonic tissues from WT or *Il21r*^-/-^ mice collected at 9 days p.i. (sampled at times indicated during the 180 hr cultures). Data are the Mean ± SEM of one experiment from two independent experiments with a total of 10 (*Il21r*^-/-^) or 10 (WT) mice/group. **C**. Bacterial burden in the feces of *C*. *rodentium*-infected *Ifnar*^-/-^ versus WT controls as shown by colony forming units (CFU)/g feces. **D**. Bacterial infection kinetics in the feces and **E**. changes in body weight of *Ifng*^-/-^ mice versus WT controls following infection with *C*. *rodentium*. The dashed line represents the sensitivity of the culture method. The results are the Mean ± SEM of 5 mice per group. *p < 0.05 determined by Mann-Whitney *U* test. **F**. Intracellular expression of IFN-γ by CD4^+-^ T cell isolated from the distal colon LP of *Il21r*^-/-^ mice or WT controls with (left panel) or without (right panel) PMA/ionomycin stimulation. **G**. The kinetics of the intracellular expression of IFN-γ by ovalbumin-specific mucosal CD4^+^ T cells in WT mice at 3, 7, 9, and 14 days after OVA-*C*. *rodentium* infection, as determined by flow cytometry. **H-K**. The expression of IFN-γR1 and IFN-γR2 (CD119) by CD4^+^ T cells isolated from the LP of the distal colon of uninfected *Il21r*^-/-^ and WT mice (*n* = 7/genotype) and infected mice 9 days p.i. (*n* = 4/genotype). Data are the Mean ± SEM of percentage of CD119^+^CD4^+^ T cells, with statistical significance determined by Mann-Whitney *U* test.

The absolute requirement of CD4^+^ T cell responses during *C*. *rodentium* infection has been reinforced by the observations that CD4^+^ T cell-deficient mice (CD4^-/-^), but not CD8^+^ T cell-deficient mice (*β*_*2*_*m*^-/-^), showed greater susceptibility to *C*. *rodentium*-induced colitis and increased systemic dissemination of the bacterium to extra-intestinal sites [16]. Consistent with our findings that IFN-γ, but not IFN-α, was the dominant interferon expressed in the colon after infection with *C*. *rodentium* (Fig 4A), significantly lower concentrations of IFN-α than IFN-γ were detected in the colon of both infected WT and *Il21r*^-/-^ mice (Fig 4B). Collectively, these findings suggested that IFN-γ is the predominant interferon produced in the colon in response to *C*. *rodentium* and that the lack of an intact IL-21/IL-21R signaling axis does not negatively affect the IFN-γ expression and production in response to infection in *Il21r*^-/-^ mice.

Considering that both type I- and type II-specific ISGs were impaired in infected *Il21r*^-/-^ mice, we investigated which type of interferon was required for the control of infection. Type I interferons have been studied mostly in the context of viral infections [7]. The roles played by type I interferons in non-viral infections, including bacterial infections have recently been investigated [7, 27, 28]. We further experimentally determined roles played by type I interferons in host protection following *C*. *rodentium* infection. Consistent with these results, mice deficient in interferon-α/β receptor (*Ifnar*^-/-^) had bacterial burdens comparable to those of their WT controls and were able to efficiently clear infection after oral challenge (Fig 4C). However, mice deficient in IFN-γ (*Ifng*^-/-^) showed a delayed clearance similar to that seen in *Il21r*^-/-^ mice and exhibited significant weight loss early in the course of *C*. *rodentium* infection (Fig 4D and 4E). These findings suggested that type I IFNs played minimal, if any, roles in protection against *C*. *rodentium* infection in mice and that the type II IFN (i.e. IFN-γ) contributed significantly to host protection.

### IL-21/IL-21R signaling axis is not required for optimal production of IFN-γ following colon infection

The earliest step at which IL-21 could influence production of ISGs is in the production of IFN-γ itself, so we addressed these levels in the *Il21r*^-/-^ mice. It has been shown by other investigators that *Il21r*^-/-^ mice express significantly higher levels of IFN-γ in the colon LP compared with WT counterparts during dextran sulfate sodium (DSS)-induced colitis [29]. Our findings demonstrated that IFN-γ was the dominant interferon, mainly expressed by CD4^+^ T cells. However, dendritic cells, to a much lesser extent, expressed IFN-β, but not IFN-α or IFN-λ or much IFN-γ (Fig 4A). Consistent with these findings, by using an *ex-vivo* organ culture system we found that the lack of the IL-21/IL-21R signaling axis did not negatively affect the production of IFN-γ in the distal colon of *Il21r*^-/-^ mice, as evidenced by significantly higher levels of IFN-γ production in the distal colon as compared with WT controls 9 days after *C*. *rodentium* infection (Fig 4B). Although the expression of IFN-γ by gut-associated CD4^+^ T cells isolated from *Il21r*^-/-^ mice was impaired (~3 fold) as compared with WT controls, higher expression of the cytokine by NK cells (~3 fold) could explain higher levels of IFN-γ observed in the whole distal colon of the *Il21r*^-/-^ mice (S9 Fig). Likewise, significantly higher levels of IL-17A were noted in the distal colon of *Il21r*^-/-^ mice as compared with WT controls at that time (Fig 4B). This contrasts with the poorer control of the infection, which should benefit from increased IFN-γ and IL-17. No significant differences were observed in the intracellular expression of IFN-γ by CD4^+^ T cells isolated from the colonic LP of either naïve *Il21r*^-/-^ mice or naïve WT controls (Fig 4F). Intracellular staining for IFN-**γ** expression by ovalbumin-specific mucosal CD4^+^ T cells in WT mice after OVA-*Citrobacter* infection demonstrated that CD4^+^ T cells are a major source of *Citrobacter*-induced IFN-**γ** following infection in the intestine (Fig 4G). Because IFN-γ production is higher, not lower, in the colons of *Il21r*^-/-^ mice, the reduction in ISGs cannot be due to a decrease in IFN-γ itself or to the need for IL-21 signaling to optimally induce IFN-γ. Thus, the effect on ISGs must be downstream of levels of IFN-γ itself. We therefore asked whether the effect on ISGs was due to a decrease in IFN-γ receptor expression, the next step that might account for the lower levels of ISGs. The lack of the IL-21/IL-21R signaling axis did not impair the expression of IFN-γR1 and IFN-γR2 (CD119) by CD4^+^ T cells isolated from the LP of *Il21r*^-/-^ mice 9 days after *C*. *rodentium* infection as compared with WT controls (Fig 4H–4K). Indeed, CD4^+^ T cells isolated from the LP of *Il21r*^-/-^ mice expressed similar levels of IFN-γR1 and IFN-γR2 as WT controls (Fig 4H–4K). Collectively, these findings demonstrated that the IL-21/IL-21R axis was not required for optimal expression and production of IFN-γR β-chain and IFN-γ and that the increased bacterial burden in *Il21r*^-/-^ mice and reduced expression of ISGs were not due to impairment of IFN-γ or its receptor in these mice, but must be further downstream in the IFN-γ signaling pathway.

### IL-21 is required for optimal activation of STAT1 in CD4^+^ T cells

**Fig 5:**
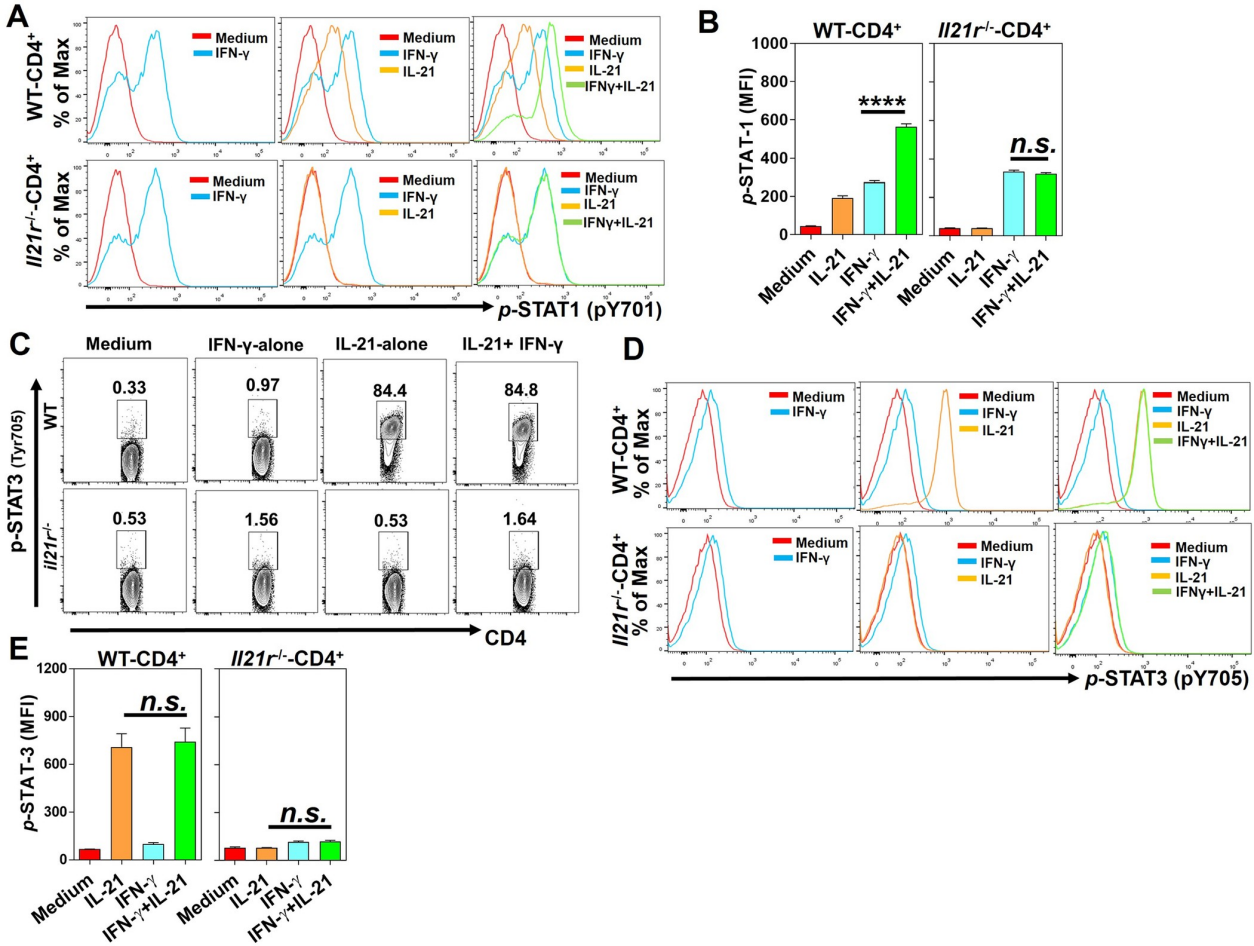
IL-21 is required for optimal activation of STAT1 in CD4^+^ T cells. **A**. Flow cytometric analysis of phosphorylated (*p*-) STAT1 (*p*Y701) in CD4^+^ T cells isolated from WT (top row) and *Il21r*^-/-^ (bottom row) mice in the presence of mIFN-γ (20 ng/ml) or mIL-21 (20 ng/ml) alone or in combination (20 ng/ml of each cytokine) for 10 min. Cells receiving no cytokine treatments were used as controls (red histograms). **B**. Bar graphs showing the average mean fluorescent intensity (MFI) increase in *p*-STAT1 in WT (left panel) and *Il21r*^-/-^ mice (right panel). **C-E.** Flow cytometry analyzing phosphorylated (*p*-) STAT3 (*p*Y705) in CD4^+^ T cells isolated from WT (top row) and *Il21r*^-/-^ (bottom row) mice in the presence of mIFN-γ (20 ng/ml) or mIL-21 (20 ng/ml) alone or in combination (20 ng/ml of each cytokine) for 30 min. Cells receiving no cytokine treatments were used as controls (red histograms). Bar graphs showing the average mean fluorescent intensity (MFI) increase in *p*-STAT3 in CD4^+^ T cells isolated from WT (left panel) and *Il21r*^-/-^ mice (right panel). The results are the Mean ± SEM of one experiment (*n* = 3/genotype/condition) from two independent experiments. *n*.*s*, not significant; One-way ANOVA followed by Bonferroni post-hoc adjustment test for multiple comparison.

If the IL-21/IL-21R axis is not necessary for expression of IFN-γ or its receptor, we asked whether it affected the next step in signal transduction downstream of the IFN-γR, STAT1 phosphorylation. It is known that IL-21 signals mainly via STAT3 but also via STAT1 and STAT5 [21, 30, 31]. We therefore hypothesized that IL-21 acts in concert with IFN-γ to facilitate the activation of STAT1 in CD4^+^ T cells and that might explain why *Il21r*^-/-^ mice failed to optimally express multiple ISGs in the distal colon after *C*. *rodentium* infection. To address this hypothesis, we stimulated total splenocytes with either recombinant mIL-21 or mIFN-γ alone or in combination and analyzed the activation of STAT proteins by CD4^+^ T cells. Interestingly, a combination of mIL-21 and mIFN-γ (20 ng/ml of each cytokine) led to significantly enhanced phosphorylation of STAT1 in CD4^+^ T cells, as compared with CD4^+^ T cells stimulated with either mIL-21 or mIFN-γ alone (Fig 5A, top row, Fig 5B). As expected, the stimulation of CD4^+^ T cells isolated from *Il21r*^-/-^ mice with a combination of mIL-21 and mIFN-γ did not enhance the activation of STAT1, as compared with cells stimulated with mIFN-γ alone (Fig 5A, bottom row, Fig 5B). However, no significant differences were observed between the activation of STAT1 in CD4^+^ T cells stimulated by mIFN-γ alone in the two genotypes, indicating that the IFN-γ axis is functional in the absence of IL-21/IL-21 R signaling in *Il21r*^-/-^ mice. However, the treatment of splenocytes with a combination of IFN-γ with IL-17A or IL-22 did not result in enhanced activation of STAT1 in CD4^+^ T cells isolated from WT or *Il21r*^-/-^ mice (S10A–S10D Fig). These findings suggest that IL-21, in collaboration with IFN-γ, enhances the expression of ISGs by CD4^+^ T cells via enhanced phosphorylation of STAT1, but that IL-21/IL-21R signaling axis is not necessary for IFN-γ to induce the phosphorylation of STAT1. In addition, we next investigated other possible complementary mechanisms besides a direct effect of IL-21 on STAT1.

It is known that the IL-21 binding to its cognate receptor, IL-21R, leads to the activation of STAT3 protein and subsequently activates a transcription program that includes some IL-21 target genes [31]. Remarkably, some of the IL-21 target genes (i.e. *Gzma*, *Gzmb*, *Il10*) are known ISGs with non-redundant critical roles in host protection against a wide variety of microbial pathogens, including enteric infections [30]. Based on this, we analyzed the activation of STAT3 following stimulation with a mIL-21 or mIFN-γ alone or in combination. Although phosphorylation of STAT3 was induced in CD4^+^ T cells by the IL-21 stimulation alone, the combined application of IL-21 and IFN-γ did not further enhance STAT3 phosphorylation (Fig 5C, Fig 5D, top row, and S11 Fig). As expected, CD4^+^ T cells isolated from *Il21r*^-/-^ mice failed to induce the STAT3 activation upon treatment with IL-21 alone or in combination with IFN-γ (Fig 5C and Fig 5D, bottom row, Fig 5E). To test whether STAT3 played any role in the collaboration observed between IL-21 and IFN-γ in ISG induction, we generated conditional knockout mice.

### Lack of STAT3 signaling in CD4^+^ T cells renders the host susceptible to enteric infection

**Fig 6:**
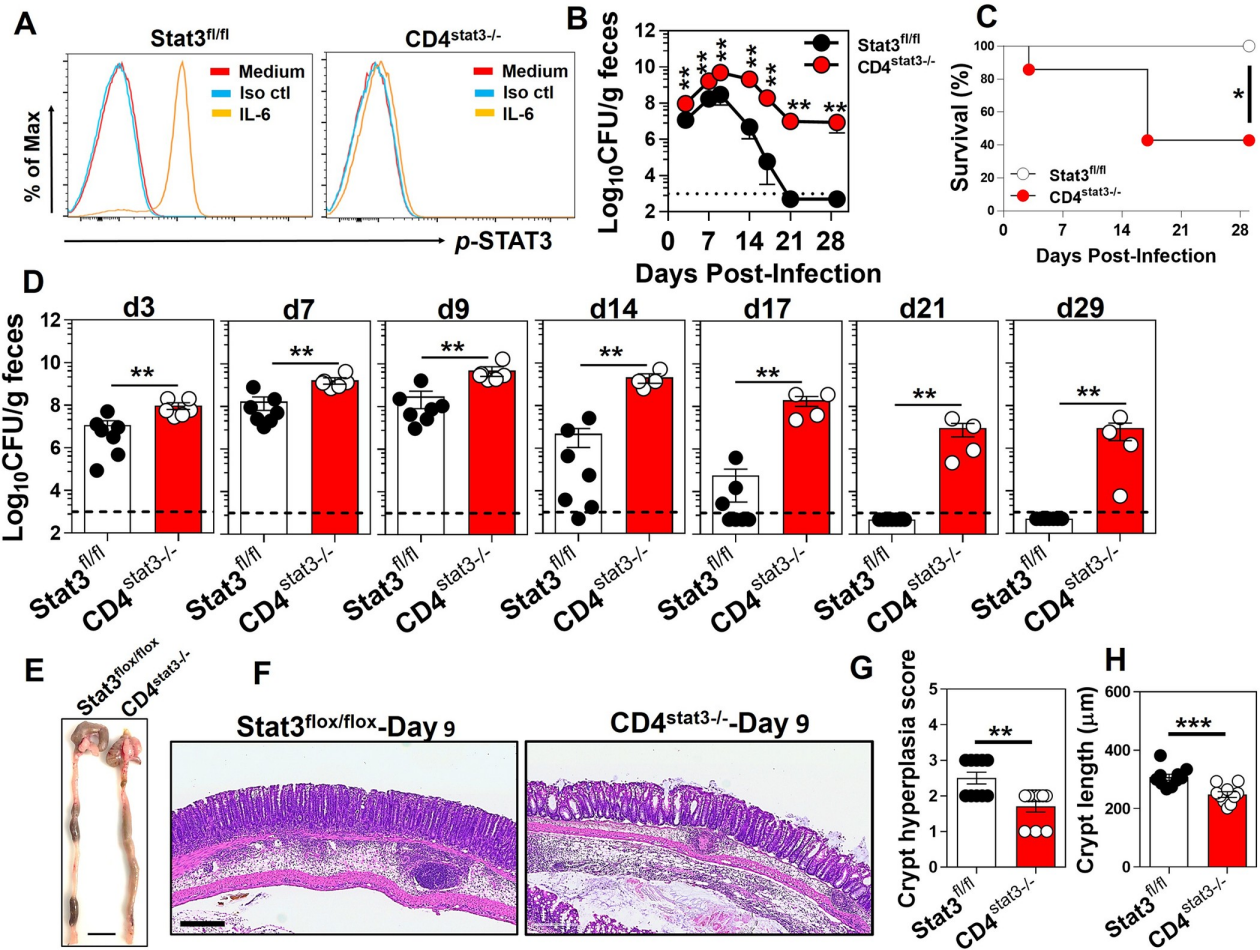
Lack of STAT3 signaling in CD4^+^ T cells renders the host susceptible to enteric infection. **A**. IL-6 stimulation (20 ng/ml) of splenocytes from CD4^stat3-/-^ mice did not induce the phosphorylation of STAT3 protein, indicating the complete abrogation of the STAT3 signaling in the conditional KO CD4^+^ T cells. **B**. Infection kinetics and **D**. bacterial burden in the feces of mice with conditional deletion of STAT3 in CD4^+^ T cells (CD4^stat3-/-^) and littermate controls (STAT3^flox/flox^) shown as colony forming unit (CFU)/g feces. The dashed line represents the sensitivity of the culture method. **p < 0.01 determined by Mann-Whitney *U* test. **C**. CD4^stat3-/-^ mice exhibited increased susceptibility to colon infection upon inoculation with 5×10^8^ CFU of *C*. *rodentium*. Survival is represented by Kaplan-Meier survival curves (*n* = 4–7 mice/group). *p < 0.05. The results are the Mean ± SEM of 4–7 mice per group from one independent experiment. Mice with STAT3 selectively deleted in CD4^+^ T cells are contrasted with littermate mice with a floxed STAT3 gene but without the Cre gene to delete the floxed gene. **E.** Representative images of gross pathology in the colon of CD4^stat3-/-^ mice and Stat3^flox/flox^ mice infected with *C*. *rodentium* for 9 days. The scale bar, 1 cm. **F.** Representative images of microscopic pathology in the distal colon of CD4^stat3-/-^ mice and STAT3^flox/flox^ controls 9 days after infection with *C*. *rodentium*, as assessed by hematoxylin and eosin (H&E) staining (5×). The scale bar, 300 μm. **G, H**. Crypt hyperplasia scores and crypt length (μm) in the distal colon of CD4^stat3-/-^ mice and STAT3^flox/flox^ controls at 9 days after infection with *C*. *rodentium*. Data are the Mean ± SEM from two pooled independent experiments using CD4^stat3-/-^ (*n* = 10) or STAT3^flox/flox^ (*n* = 10) mice. *p < 0.05 determined by Mann-Whitney *U* test.

The biological functions of IL-21 are mediated mainly via the activation of the STAT3 signaling axis downstream of the IL-21R in a wide variety of hematopoietic cells, although IL-21 is also known to exert some biological effects via the activation of STAT1 and STAT5 [23]. It has been shown that STAT3 is activated in intestinal epithelial cells following *C*. *rodentium* infection *in vivo* and that mice conditionally deficient in STAT3 in epithelial cells (Stat3^ΔIEC^) were highly susceptible to infection and developed severe colitis after infection with *C*. *rodentium* [32]. Our findings suggested that IL-21 was exclusively expressed by mucosal CD4^+^ T cells and that mucosal CD4^+^ T cells expressed higher levels of transcripts for IL-21R than other mucosal cells tested, although several immune cell types express this receptor (Fig 2B and 2C). We bred CD4-conditional STAT3^-/-^ mice by crossing CD4-Cre mice with STAT3^flox/flox^ mice (See Materials and Methods). The abrogation of the STAT3 signaling pathway in these mice was confirmed by the lack of STAT3 activation upon IL-6 treatment of CD4^+^ T cells isolated from CD4^stat3-/-^ mice (Fig 6A). To address the role played by the IL-21/IL-21R signaling axis in CD4^+^ T cells in host protection after *C*. *rodentium* infection, we assessed the bacterial burden, the infection kinetics and survival rates in conditional knockout mice with a CD4^+^ T cells-specific deletion of STAT3 activity (Fig 6B–6D). The CD4^stat3-/-^ conditional deficient mice showed increased bacterial burden at early time points (days 3 and 7), a higher peak bacterial load at day 9 p.i., and impaired clearance at days 14, 17, 21 and 29. Thus, they were even more impaired in their ability to handle *C*. *rodentium* infection than the *Il21r*^-/-^ mice. That may be because STAT3 is critical not only for IL-21 signaling, but also for IL-17, and induction of T_H17_ cells, which are another key mediator of *C*. *rodentium* clearance. Survival of these mice after *C*. *rodentium* infection was also significantly impaired (Fig 6C). These finding are consistent with previous reports demonstrating that the STAT3 activation in T_H17_ and T_H22_ CD4^+^ T cells is important for protection against *C*. *rodentium* [33]. This confirms that a deficiency in these cytokines in CD4^+^ T cells alone is sufficient to seriously impair their ability to handle this bacterial colonic infection. At necropsy, the conditional deletion of STAT3 signaling in CD4^+^ T cells resulted in watery stool as well as hematomas along the lengths of the distal colons of CD4^stat3-/-^ mice 9 days after *C*. *rodentium* infection (Fig 6E). Histological examinations of the distal colons of mice 9 days after infection demonstrated significantly lower crypt hyperplasia scores, a hallmark of pathology during *C*. *rodentium* infection, and considerably shorter crypt lengths in of CD4^stat3-/-^ mice (Fig 6F–6H). Collectively, these findings indicated that *C*. *rodentium* infection induced moderate pathological changes in CD4^stat3-/-^ mice as compared with littermate STAT3^flox/flox^ control mice, despite significantly higher bacterial burdens in the distal colons of those mice.

### STAT3 signaling is not required for the IL-21-induced optimal expression of ISGs in CD4^+^ T cells

**Fig 7:**
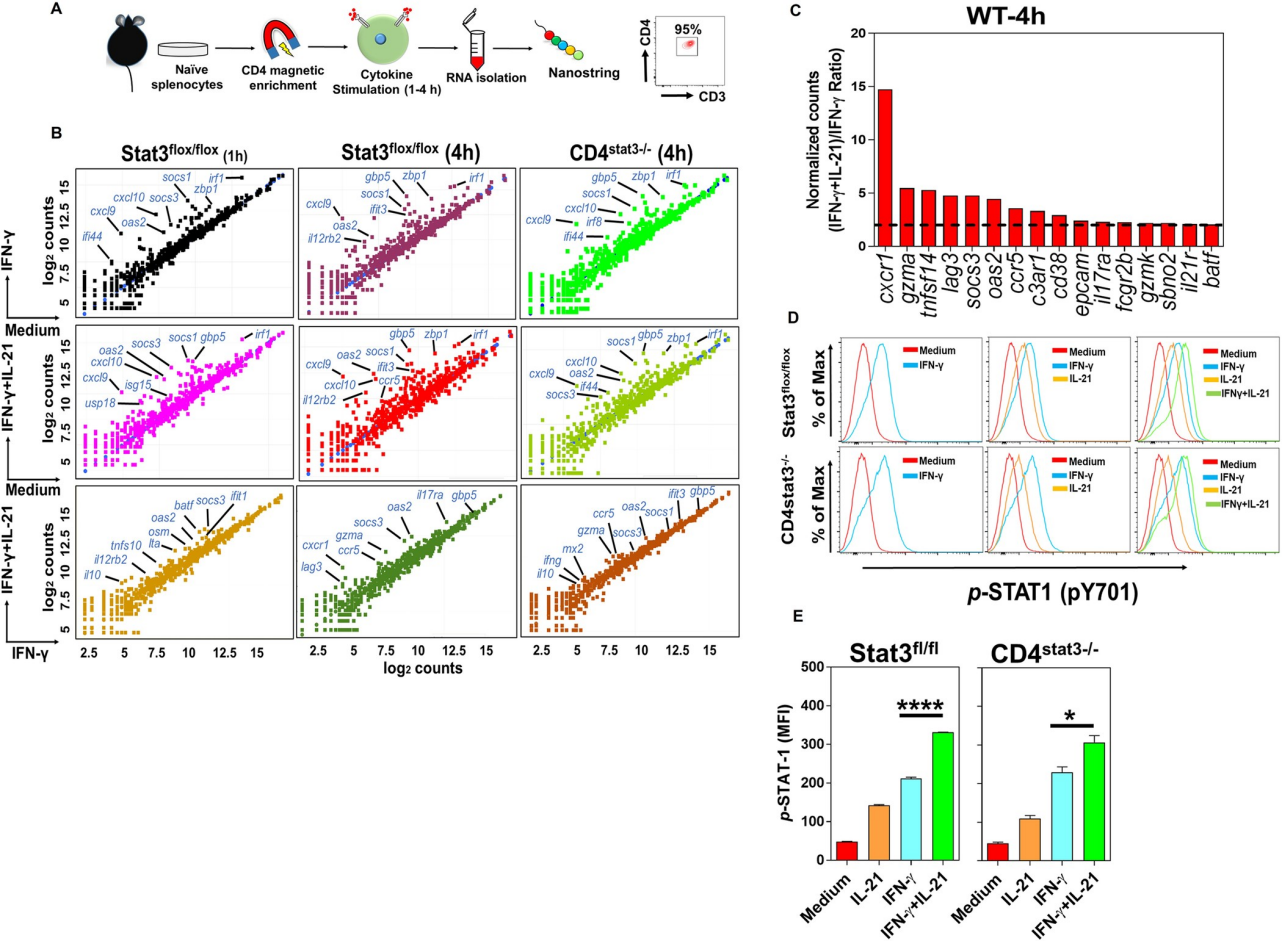
STAT3 signaling is not required for the optimal expression of ISGs by IL-21 and IFN-γ collaboration in CD4^+^ T cells. **A**. Experimental setup and cell purity after CD4^+^ T cell negative selection. **B**. Scatter plot representation (log_2_ values) of gene expression signatures in pooled purified naïve CD4^+^ T cells from STAT3^flox/flox^ (*n* = 7) or CD4^stat3-/-^ (*n* = 5) mice after treatment with rmIFN-γ or rmIFN-γ+IL-21 vs. medium (top and middle row, respectively) or rmIFN-γ plus rmIL-21 vs. rmIFN-γ alone (bottom row; 20 ng/ml of each cytokine) for 1–4 hr. Purified cells receiving no cytokines were used as controls. **C**. Histogram shows fold increase of genes in rank order in WT CD4^+^ T cells following stimulation with single or dual cytokines. Genes with fold difference ≥ 2 between (IFN-γ+IL-21)/IFN-γ were considered. **D**. Flow cytometric analysis and **E**. MFI measurements of phosphorylated (*p*-) STAT1 (*p*Y701) in CD4^+^ T cells isolated from STAT3^flox/flox^ (top row) and CD4^stat3-/-^ (bottom row) mice in the presence of mIFN-γ (20 ng/ml) or mIL-21 (20 ng/ml) alone or in combination (20 ng/ml of each cytokine) for 30 min. Cells receiving no cytokine treatments were used as controls (red histograms). The results are the Mean ± SEM of 3 mice per group from one experiment out of two independent experiments. *p < 0.05; ****p < 0.0001; One-way ANOVA followed by Bonferroni post-hoc adjustment test for multiple comparison.

It is known that IL-21 exerts some of its downstream effects via the activation of STAT1, STAT3 and STAT5 [21], whereas the induction of IFN-γ-target genes exclusively requires the activation of STAT1 signaling pathway [34]. We already have shown that the activation of STAT3 in CD4^+^ T cells did not increase when stimulated with a combination of IL-21 and IFN-γ compared with cells treated with IL-21 alone (Fig 5C and 5D). Hypothetically, the increased STAT1 phosphorylation when IL-21 was combined with IFN-γ could have been due to a direct effect of IL-21 on STAT1, or to an indirect effect of some events downstream of IL-21’s principal signaling pathway through STAT3. Thus, we sought to determine whether the optimal expression of ISGs requires intact STAT3 signaling in CD4^+^ T cells or is occurring in a STAT3-independent manner. To specifically target STAT3 in CD4^+^ T cells and to avoid the off-target effects of the global STAT3 deletion and its possible indirect effects of these on CD4^+^ T cells, we stimulated conditional *Stat3*^-/-^ CD4^+^ T cells (lacking STAT3 only in CD4^+^ T cells) or control CD4^+^ T cells with IFN-γ or IL-21 or a combination of both and determined the expression of ISGs following the single or dual cytokine treatment. In particular, we sought to determine whether the optimal expression of ISGs by CD4^+^ T cells requires an intact STAT3 signaling or that the enhanced expression of ISGs in CD4^+^ T cells following treatment with both IFN-γ and IL-21 occurs in a STAT3-independent manner. Further analyses demonstrated that *Stat3*^-/-^ CD4^+^ T cells upregulated an ISG profile in response to exogenous IFN-γ or IFN-γ+IL-21 in a fashion similar to CD4^+^ T cells from STAT3^flox/flox^ controls (Fig 7B, top and middle rows). Accordingly, *Stat3*^-/-^ CD4^+^ T upregulated a gene signature profile very similar to the one induced in control animals in response to a combination of rmIFN-γ and IL-21 (Fig 7B, bottom row, and Fig 7C). These findings show that the lack of a functional STAT3 in CD4^+^ T cells does not impair the expression of ISGs in *Stat3*^-/-^ CD4^+^ T cells and that both *Stat3*^-/-^ CD4^+^ T cells and cells from STAT3^flox/flox^ littermates upregulated analogous gene profiles in response to IFN-γ and IL-21. Furthermore, the lower panels comparing the cytokine combination with IFN-γ alone show that many ISGs are upregulated more by the combination than by IFN-γ alone in both the intact and STAT3-deficient CD4^+^ T cells. We conclude that the enhanced induction of most ISGs when IL-21 is added to IFN-γ does not depend on the former’s signaling through STAT3.

### Enhanced activation of STAT1 in CD4^+^ T cells by IL-21 is independent of STAT3

To more mechanistically delineate the roles played by IL-21 in enhanced expression of ISGs via the facilitated activation of STAT1, we particularly asked whether the enhancement of STAT1 phosphorylation by combining IL-21 with IFN-γ was a direct effect of IL-21 on STAT1 or an indirect effect dependent on STAT3. To address this question, we measured STAT1 activation in *Stat3*^-/-^ CD4^+^ and *Stat3*^+/+^ CD4^+^ T cells in response to IFN-γ or IL-21 alone or in combination. Our findings indicated that *Stat3*^-/-^ CD4^+^ T cells and control *Stat3*^+/+^ CD4^+^ T cells significantly and comparably phosphorylated STAT1 more in response to a combination of IFN-γ and IL-21 than to the single-cytokine-treated CD4^+^ T cells (Fig 7D and 7E). These findings suggest that IL-21-induced enhancement of STAT1 activation was independent of STAT3. Interestingly, the IL-21 treatment of CD4^+^ T cells isolated from mice deficient in IFN-γ/IFN-γR signaling (*Ifngr*^-/-^) induced the activation of STAT1 in these cells, indicating that the endogenous IFN-γ signaling pathway is not required for the STAT1 activation by IL-21 (S12A Fig). Moreover, the expression of LAG-3 in CD4^+^ T cells isolated from *Ifngr*^-/-^ mice was not induced by IFN-γ treatment alone, and was not higher with combined IL-21/IFN-γ than with IL-21 alone, indicating the expression of LAG-3 is not enhanced by IL-21 in the absence of an intact IFN-γ/IFN-γR signaling pathway (S12B Fig).

## Discussion

In this study we discovered a previously unknown collaboration between IL-21 and IFN-γ inducing interferon-stimulated genes (ISGs), in the course of studies on the role of the IL-21/IL-21R signaling pathway in resistance to and clearance of infection with a minimally-invasive murine intestinal pathogen *C*. *rodentium*. Deficiency in this pathway leads to attenuated inflammation in the colon following infection with this pathogen and impaired clearance of the pathogen. These effects appear to be partially dependent on ISGs, even though IL-21-induced STAT3 activation could play a role in IL-17- or IL-22-mediated protection against *C*. *rodentium*. Nanostring analysis identified that the majority of genes substantially impaired (≥ 2-fold) in the whole distal colon of *Il21r*^-/-^ mice as well as in CD4^+^ T cells isolated from those animals after infection with *C*. *rodentium* were ISGs. We showed that IFN-γ, but not IFN-α/β, mediated resistance to and clearance of *C*. *rodentium*. Importantly, we have discovered unexpectedly that IFN-γ collaboratively interacts with IL-21 for the optimal activation of STAT1 and subsequent induction of ISGs. Thus, the collaboration occurs at the level of STAT1 downstream of the IFN-γR, not in the expression of IFN-γ itself or its receptor. We further showed the absolute requirement of the STAT3 signaling pathway in CD4^+^ T cells for host defense against *C*. *rodentium*, by a separate mechanism because the enhanced activation of STAT1 and the subsequent induction of ISGs in CD4^+^ T cells by the combination of IL-21 and IFN-γ occurred in a STAT3-independent manner. STAT3 is known to be critical for IL-17 function which is important for host defense against extracellular pathogens [33].

The expression of ISGs is tightly regulated by the immune system to avoid excessive and persistent induction causing inflammation and tissue damage [35]. Excessive expression of ISGs has been linked to several inflammatory conditions [34]. Multiple microbial pathogens trigger type-specific interferons, resulting in the transcription of a wide range of downstream gene signatures with distinct or overlapping functions. Some of these genes have roles in the regulation of immunity and inflammation in different immune compartments, including the colon [36–38]. Several ISGs play important roles in host defense against viral, bacterial, and parasitic infections by directly targeting genes conferring protection against infection [39]. Mice deficient in *Isg15* (*Isg15*^-/-^) are more susceptible to infections with several viral [40, 41] and bacterial infections [42]. As such, studies *in vivo* showed that IFN-β produced by *Legionella*-infected macrophages promoted host defense via the upregulation of ISGs and this induction was required for host defense against *L*. *pneumophila* [43].

In addition to protective roles, ISGs can mediate inflammatory responses in the colon, predisposing the host to colitis-associated colon cancer [44]. Similarly, IL-21 was highly expressed in the colons of C57BL/6 mice with dextran sulfate sodium (DSS)-induced colitis and *Il21r*^-/-^ mice manifested milder DSS-induced colitis as compared with their WT counterparts [29]. These findings suggest that the IL-21/IL-21R signaling axis could be part of a positive feedback loop that amplifies an inflammatory response in the gut. Our data support this interpretation, because the *Il21r*^-/-^ mice had less inflammation and edema in the distal colons after *C*. *rodentium* infection despite having a higher bacterial burden (Fig 1C).

A network of cytokines signals through the STAT3 pathway with overlapping or opposing pro- or anti-inflammatory properties, including IL-6, IL-10, IL-17, IL-21 and IL-22. These cytokines activate the STAT3 signaling cascade by phosphorylation and subsequent STAT3 dimerization and translocation into the nucleus [45, 46]. In a mouse model of intestinal inflammation (DSS-induced colitis), for example, IL-22 was excessively secreted and the antibody blockade of IL-22 led to exacerbated inflammation in the colon, whereas the IL-22 overexpression resulted in attenuated inflammation [45]. These results suggest anti-inflammatory roles for IL-22 in the colon following intestinal inflammation. IL-6 is historically considered a pro-inflammatory cytokine and is known to promote inflammation in several models of inflammatory disease [46]. Conversely, the genetic loss of IL-6 or the antibody blockade of IL-6 results in attenuated colitis following intestinal inflammation [47]. While STAT3 plays an important role in protection against *C*. *rodentium* infection as we have seen, we found that it is not necessary for the collaboration between IL-21 and IFN-γ.

Minimally-invasive enteric microbial pathogens adhere very closely to the intestinal epithelial surfaces and induce drastic physiological and cytoskeletal changes and structural reorganization in underlying epithelial cells [48]. Several defensive mechanisms have evolved to control enteric microbial infections at the intestinal epithelial barrier [49]. A cooperation between innate lymphoid cell (ILC)-derived IL-22 and IFN-λ was required for the optimal expression of STAT1 in IECs, leading to enhanced expression of ISGs by these cells and subsequent control of rotavirus infection *in vivo* [6]. It has been suggested that an evolutionary collaboration between two distinct but related cytokine signaling pathways facilitates the control of infection in the intestine. Our finding demonstrated that the receptor for IL-21 was more highly expressed by mucosal CD4^+^ T cells during intestinal infection than by other immune subsets in the colon LP (i.e. dendritic cells, macrophages, neutrophils, NK cells) and that mucosal CD4^+^ T cells expressed significant levels of transcripts for the IFN-γ receptor. Concurrent engagement of both receptors on CD4^+^ T cells was essential for optimal induction of ISGs in those cells. Our findings also indicated that colonic IECs did not express the receptor for IL-21 in response to intestinal microbial infection, whereas IFN-γ receptor was expressed by these cells. The lack of the expression of the IL-21 receptor by IECs excludes these cells as targets for the collaborative effects of IL-21 and subsequent modulation of ISG expression during *C*. *rodentium* infection as shown in other models of intestinal infection, in which IECs were the main targets of IL-22 and IFN-λ cooperation [6].

The indispensable role of CD4^+^ T cells in protection against intestinal infection with *C*. *rodentium* has been established in several studies [16, 17]. Mice deficient in CD4^+^ T cells (but not CD8^+^ T cells) are extremely susceptible to intestinal infection with *C*. *rodentium* as well as to the systemic spread of the bacterium to extra-intestinal sites, including the mesenteric lymph nodes (MLNs), spleen and the liver [15]. Intestinal infection with this pathogen elicits a T_H1_-biased immune response, characterized by the induction of IFN-γ and TNF-α (17). CD4^+^ T cells play a central role in immune response to intestinal infection with *C*. *rodentium* via the production of cytokines required for host resistance to this pathogen, including IL-17, IL-22 and IFN-γ, and they have been shown to be a main source of antigen-specific induction of IFN-γ during intestinal infection with this bacterium [17]. Our observations show dual roles for mucosal CD4^+^ T cells following infection with *C*. *rodentium* in the colon: First, IL-21 was exclusively expressed by mucosal CD4^+^ T cells of the colon LP and second, mucosal CD4^+^ T cells express significant levels of transcripts for IL-21R and IFN-γR and are targets for the collaborative activity of IFN-γ and IL-21. These finding identify a previously unidentified collaboration between two distinct signaling pathways in the optimal expression of ISGs in CD4^+^ T cells, and protection against *C*. *rodentium*, and may further explain the absolute requirement of CD4^+^ T cells in the regulation of mucosal immune response to this pathogen in the colon.

The type I IFNs have emerged as key players in host defense against both extracellular and intracellular bacterial pathogens in recent years [7, 27]. We observed that both type I- and type-II specific ISGs were induced in the distal colon following infection with *C*. *rodentium*. Further analysis demonstrated that mice deficient in IFN-α/β signaling (*Ifnar*^-/-^) managed to clear infection with the bacterium in kinetics similar to WT controls. However, mice lacking functional IFN-γ (*Ifng*^-/-^) failed to clear the infection, indicating that type II-specific, but not type I-specific, ISGs were required for host defense in the colon.

Other effects of IL-21 receptor deficiency on intestinal host defense have been reported very recently involving defective IgA responses to atypical commensal bacteria, such as segmented filamentous bacteria (SFB) and *Helicobacter* species, indirectly affecting *C*. *rodentium*-induced immunopathology [50]. However, in this situation, the effect was mostly on inflammation and the effect on bacterial burden was minor. This is clearly distinct from our model in which it is clear that IFN-γ plays a critical role and the role of IL-21 is mainly to amplify the IFN-γ signal in CD4^+^ T cells. Furthermore, we have cohoused mice for at least 2 weeks in all experiments to equalize intestinal microbiota, as mice, which are coprophagic, rapidly equilibrate their microbiomes when cohoused for 2 weeks [51–54], and have replicated the same results with *Il21r*^-/-^ mice vs. heterozygous controls that were bred from the same group of *Il21r*^-/-^ mothers, to further ensure that the microbiota were equivalent (S1 Fig).

The collaborative link we established here between IFN-γ and IL-21 signaling pathways provides mechanistic elucidation to the enduring conundrum as to why the lack of an intact IL-21/IL-21R signaling axis renders host susceptible to a wide range of pathogens [55, 56]. It also provides insight into why the lack of an intact IL-21/IL-21R pathway leads to attenuated inflammation in the colon following insults during infectious and non-infectious insults [29, 44]. Considering that IECs, as the first line of defense against minimally-invasive intestinal pathogens including *C*. *rodentium*, do not express the receptor for IL-21, our findings suggest the collaborative effect we describe between IFN-γ and IL-21/IL-21R signaling axes acts deep in the lamina propria of the colon as a second defensive layer against mucosal pathogens. Understanding the interactions of these cytokine networks and their signaling pathways should allow development of novel therapeutic targets for colonic infections, inflammatory bowel disease, and promotion of mucosal vaccine efficacy.

## Materials and methods

### Animals

Six- to eight-week old sex- and age-matched female mice were used in all experiments. The mice were bred in-house or purchased from the Jackson Laboratory (Bar Harbor, ME, USA). In order to exclude the effects of differing microbiome compositions between mouse genotypes on the experimental designs, the knockout and WT mice (both on a C57BL/6 background) were cohoused for 2 weeks at a 1:1 ratio before all experiments as described before [51–54]. In addition, to further exclude microbiome differences, we bred WT or *Il21r*^-/-^ males to the same group of *Il21r*^-/-^ females to produce matched pups that were either heterozygotes (WT phenotype) or homozygous *Il21r*^-/-^ and that were then foster nursed together, so the microbiome obtained from their mothers and nursing mothers would be identical (S1 Fig). C57BL/6 were purchased from Charles River Laboratories (Wilmington, MA, USA). B6N.129-*Il21r*^*tm1Kopf*^/J (019115) were purchased from the Jackson Laboratory. *Ifnar*^-/-^ and *Ifngr*^*-/-*^ mice on a C57BL/6 background were kindly provided by Dr. Howard Young (NCI/NIH).

We generated mice in which STAT3 protein is conditionally deleted in CD4^+^ T cells (CD4^stat3-/-^) by breeding CD4-Cre mice (Tg(Cd4-cre)1Cwi/BfluJ; 017336) to STAT3^flox/flox^ (B6.129S1-*Stat3*^*tm1Xyfu*^/J mice; 016923 (both strains from the Jackson Laboratory) as described earlier [57]. Mice were genotyped using DNA isolated from tail snips. STAT3^flox/flox^ littermates were used as wild-type (WT) controls.

### Ethics statement

All experiments were carried out in accordance with guidelines and protocols approved by the National Cancer Institute Animal Care and Use Committee in compliance with the National Institutes of Health Guidelines (VB-014).

### Cell cultures and cytokines

Spleens were aseptically removed from naïve WT, *Il21r*^-/-,^ STAT3^flox/flox^ or CD4^stat3-/-^ mice and cells were mechanically disrupted through a 100 μm cell strainer (BD Biosciences, San Jose, CA) using the plunger of a 6 ml syringe. RBCs were lysed in ACK lysing buffer (Lonza, Walkersville, MD), and the remaining cells were washed twice with ice-cold PBS. A total of 5×10^6^ splenocytes were cultured in duplicate in 1 ml of RPMI 1640 (Gibco) supplemented with 10% FBS and 100 μg/ml penicillin/streptomycin (all from Gibco). Cells were allowed to rest for 2 additional hours at 37°C and subsequently were stimulated in the presence of recombinant murine IFN-γ, IL-17A, IL-21 and IL-22 (20 ng/ml; Peprotech, Rocky Hill, New Jersey, USA) alone or in combination (IFN-γ+IL-17A; IFN-γ+IL-21; IFN-γ+IL-22; 20 ng/ml each). Cells receiving no cytokine treatments were used as controls. Cells were harvested at different intervals following the addition of cytokines and were stained with indicated antibodies.

### Infection protocol and quantification of bacterial burden

*C*. *rodentium* strain DBS100 (ATCC 51459) was propagated in Luria-Bertani (LB) broth at 37°C, harvested by centrifugation, and resuspended in PBS at a concentration of 5×10^9^ colony forming units (CFU)/mL. In some experiments, an ovalbumin-expressing *C*. *rodentium* (OVA-*Citrobacter*) under a kanamycin-resistance gene was used for infection. Mice infected with OVA-*Citrobacter* were given kanamycin (1g/L) in drinking water *ad libitum*, starting 4 days before infection and during the entire course of infection to prevent loss of OVA expression. Mice were infected with 100 μl of the bacterial suspension containing 5×10^8^ CFU of *C*. *rodentium*/mouse by oral gavage as described previously [58, 59]. For bacterial quantification, fecal pellets (50–100 mg) were weighed, homogenized in 2 mL of sterile PBS, serially diluted, and plated onto MacConkey agar as described before [58, 59]. The detection limit of the culture method was 10^3^ CFU/g feces.

### Histological examinations

The colons were cleaned, rolled into a Swiss roll configuration, fixed in 10% buffered formalin overnight, followed by fixation in 70% ethanol and subsequently embedded in paraffin. Tissue sections (5-μm thick) were stained with hematoxylin and eosin (H&E) and digitized with Aperio ScanScope (Aperio, Vista CA) and were analyzed using Aperio ImageScope software. The severity of colitis was assessed by an unbiased (blinded) observer using a scoring system developed previously [60].

### Organ culture and ELISA

An *ex vivo* organ culture system was used to determine the kinetics of cytokine production in the colon of mice after *C*. *rodentium* infection as described before [26]. The distal colons from infected mice at different time-points or uninfected controls were removed, washed briefly and opened longitudinally and were cultured in RPMI1640 culture medium (Gibco) supplemented with 10% FBS, and 100 μg/ml penicillin/streptomycin (Gibco). Culture supernatants were collected at different intervals post-culture and were examined for cytokine concentrations by ELISA. Mouse ELISA kits for IFN-α, IFN-γ, IL-17A, IL-21 and IL-22 (all from eBioscience) were used. Results were expressed as picograms per g tissue (pg/g).

### Colonic lamina propria (LP) lymphocyte isolation

The colonic LPLs were isolated as described elsewhere [59]. Briefly, the distal colons were removed, cut longitudinally, and washed with ice-cold PBS. The colons then were cut into small pieces and incubated for 20 min in pre-digestion solution (PBS, pH7.4, containing 30 mM EDTA and 1 mM dithiothreitol) at 37°C with agitation. After incubation, the samples were vortexed for 20 seconds and the supernatants were discarded. Subsequently, the tissues were washed twice with ice-cold PBS, minced, and digested in RPMI1640 (Gibco), containing 420 μg/ml Liberase TL (Roche, Indianapolis, IN, USA), and 0.1 mg/ml DNase (Roche), for 45 min at 37°C. Digesting tissues were further mechanically dissociated by vortexing vigorously every 10 min. Digested tissues were vortexed for an additional 20 seconds and passed through a 70-μm cell strainer (DB Falcon, San Jose, CA, USA). Isolated cells were washed twice with ice-cold PBS, counted, and stained with the indicated antibodies.

### Flow cytometry and intracellular staining

Isolated LPLs were incubated with 1 μg/10^6^ cells anti-mouse CD16/CD32 (clone 93; Biolegend, San Diego, CA, USA) in FACS buffer (PBS supplemented with 3% FBS) for 20 min on ice to block Fc receptor binding, followed by live/dead cell labeling using a LIVE/DEAD Fixable Aqua Dead Cell Stain Kit (Life Technologies, Eugene, OR, USA) for 20 min at 4°C in the dark per the manufacturer’s instructions. For surface marker staining, cells were stained in duplicate with a cocktail of the following conjugated antibodies in FACS buffer: anti-CD3-BV421 (clone 17A2), anti-CD4-Alexa Fluor 488 (clone GK1.5), anti-CD8-Alexa Fluor 700 (clone 53–6.7), anti-NK1.1-APC (clone PK136), anti-CD45 (clone 30-F11), anti-CD11b-APC (clone M1/70), anti-MHC class II (I-A/I-E)-Pacific Blue (clone M5/114.15.2), anti-F4/80-Brilliant Violet 421 (clone BM8), anti-LAG-3-PerCP/Cy5.5 (clone C9B7W), anti-EpCAM-PE-Cy7 (clone G8.8), anti-IFN-γR-β-PE (clone MOB-47; all from Biolegend), anti-CD11c-Texas Red (clone MCD11C17; Invitrogen) and LAG-3-PE (clone C9B7W; eBioscience). For measuring the surface expression of IL-21R, LPLs were stained using a biotinylated anti-IL-21R Ab (eBio4A9; eBioscience), followed by staining with PE-streptavidin (Biolegend). The immune cell number in the colon was quantified by flow cytometry using CountBright absolute counting beads according to the manufacturer’s instructions (Molecular Probes, Invitrogen). For intracellular staining, the LPLs isolated from the colon were stained without stimulation or were stimulated *ex vivo* for 8 h in the presence of PMA (50 ng/ml) and ionomycin (350 ng/ml) or chicken egg ovalbumin (OVA) (100 μg/ml; Sigma), adding brefeldin A at 10 μg/ml (all from Sigma-Aldrich) for the last 6 h. The cells then were fixed, permeabilized, and stained with anti-IFN-γ-PE (clone XMG1.2; Biolegend). Data were acquired using an LSRII flow cytometer (BD Biosciences) and were analyzed using FlowJo software (Tree Star, Inc., San Carlos, CA, USA).

### Phospho-specific flow cytometry

We applied phospho-flow cytometry analysis to investigate the phosphorylation of STAT1 or STAT3 proteins following the treatment of naïve splenocytes with a combination of recombinant murine IFN-γ, IL-17A, IL-21, IL-22 or IL-6. Following the treatment of cells (5×10^6^/well) with IFN-γ, IL-17A, IL-21 or IL-22 (20 ng/ml each) alone or in combination (IFN-γ+IL-17A; IFN-γ+IL-21; IFN-γ+IL-22; 20 ng/ml each), the cells were fixed and permeabilized followed by staining with either anti-phospho-STAT1 (pY701; clone 4a; BD Biosciences), anti-STAT3 (pY705; clone 13A3-1; Biolegend) or mouse IgG1κ isotype control (clone P3.6.2.8.1; eBioscience). Data were acquired using an LSRII flow cytometer as described earlier.

### Nanostring analysis

LPLs were isolated as described above from distal colons of naïve mice or from mice infected with *C*. *rodentium* 9 days p.i. The colonic LPL were FACS-sorted into CD4^+^ T cells (EpCAM^-^CD45^+^CD3^+^CD4^+^) by using a FACSAria cell sorter (Becton Dickinson). Splenic CD4^+^ T cells were purified (≥95% purity) using negative selection with a mouse CD4^+^ T cell isolation kit (Miltenyi Biotec, Auburn, CA) and total RNA was isolated from CD4^+^ T cells using a Qiagen RNeasy Plus Micro Kit (Qiagen, Hilden, Germany). In some experiments total RNA from whole distal colons was isolated using a Qiagen RNeasy Plus Mini Kit (Qiagen, Hilden, Germany) and used as the source of RNA for further analysis. Total RNA (100 ng) was used as samples for probe-based NanoString system (nCounter XT Code Set; Seattle, WA, USA). The raw data for each gene was compile and normalized against the spike-in positive (6 genes) and negative (8 genes) internal reference genes and were expressed as normalized counts by using the nSolver analysis software version 3.0 (NanoString Technologies). The gene expression data were further normalized to the geometric mean of the expression of internal reference genes and presented as normalized counts/gene/biological sample.

### Interferome database analysis

The status of impaired genes in the whole distal colon or CD4^+^ T cells isolated from the distal colon was tallied against the Interferome database (http://interferome.org) to establish whether the expression of a given gene is affected by type I, type II or type III interferons in the previously published databases [61]. The gene classification based on biological processes and gene ontology (GO) were performed using the Database for Annotation, Visualization and Integrated Discovery (DAVID, version 6.8; http://david.abcc.ncifcrf.gov) Bioinformatics Resources [62, 63]. The normalized Nanostring values were used to generate heatmaps by using the web-based open software Morpheus (http://software.broadinstitute.org/morpheus).

### Statistical analysis

Data were analyzed using GraphPad Prism, version 7.03, software (GraphPad, San Diego, CA, USA) and expressed as the Mean ± SEM. For statistical analyses, a 2-tailed Mann-Whitney *U* test or a one-way ANOVA followed by Bonferroni post-hoc adjustment test for multiple comparison were employed. *p < 0.05 was considered statistically significant.

## Supporting information

S1 Fig CFU in matched mice bred from *Il21r*^-/-^ mothers. WT heterozygotes (*Il21r*^-/-^) and homozygous *Il21r*^-/-^ mice were infected with *C*. *rodentium* and infection kinetics in the feces of these mice is shown as colony forming unit (CFU)/g feces. *n* = 5–8 mice/group. *p* < 0.05; *p* < 0.001 determined by Mann-Whitney *U* test.
(TIF)

S2 Fig Infection kinetics of *C*. *rodentium* in the feces (CFU/g) of mice infected with OVA-*Citrobacter* as compared with mice orally gavaged with WT-*Citrobacter*. The results are the Mean ± SEM of 5 mice/group. The dashed line represents the sensitivity of the culture method.
(TIF)

S3 Fig **A**. Gating strateg**y** for the identification of **B**. natural killer (NK) cells **D**. neutrophils and **F.** macrophages in the lamina propria of the colon following infection with *C*. *rodentium*. *Il21r*^-/-^ mice and WT controls had comparable percentages (left panels) and absolute numbers (right panels) of those cells in the lamina propria of the whole colon following *C*. *rodentium* infection **C, E, G**.
(TIF)

S4 Fig IL-21R is not expressed by the colon carcinoma cell line (MC-38) model of colonic IECs. Flow cytometric analysis of surface expression of IL-21R by MC-38 carcinoma cells.
(TIF)

S5 Fig Heatmap of gene expression profiles in the whole distal colon of uninfected and infected WT and *Il21r*^-/-^ mice at 9 days p.i., as determined by Nanostring analysis (red, upregulated; blue, downregulated). (*n* = 3/genotype/time-point).
(TIF)

S6 Fig The intracellular expression of granzyme A by splenic CD4^+^ following treatment with IL-21 or IFN-γ alone or a combination of IL-21 and IFN-γ for 24 hr. The dashed line in the graph represents the granzyme A expression in untreated splenic CD4^+^ T cells.
(TIF)

S7 Fig **A.** The flow cytometric analysis of the surface expression of IL-21R or IFN-γR by mucosal CD4^+^ T cells and **B.** CD4^+^ T cells positive for both IL-21R and IFN-γR enzymatically isolated from the colonic LP of naïve (uninfected) C57BL/6 mice (*n* = 3).
(TIF)

S8 Fig The flow cytometric analysis of the surface expression of LAG-3 by mucosal CD4^+^ T cells in the colonic LP of naïve (uninfected) WT and *Il21r*^-/-^. Data are the Mean ± SEM from two pooled independent experiments with a total of 7 (*Il21r*^-/-^) or 7 (WT) mice/group. **p < 0.001 determined by Mann-Whitney *U* test.
(TIF)

S9 Fig The expression of IFN-γ by hematopoietic and non-hematopoietic (IECs) cells isolated from the distal colons of WT and *Il21r*^-/-^ mice. Cells were isolated enzymatically from the distal colons of mice infected 9 days p.i and were FACS-sorted. The expression of IFN-γ was measured by Nanostring at described in the Methods.
(TIF)

S10 Fig **A-D.** The treatment of splenocytes with a combination of IFN-γ with IL-17A or IL-22 did not result in enhanced activation of STAT1 in CD4^+^ T cells isolated from WT and *Il21r*^-/-^ mice. The results are the Mean ± SEM of one experiment (*n* = 3/genotype/condition) from two independent experiments. *n*.*s*, not significant; One-way ANOVA followed by Bonferroni post-hoc adjustment test for multiple comparison.
(TIF)

S11 Fig The kinetics of STAT3 phosphorylation following stimulation with recombinant murine IL-21. Naïve splenocytes from WT animals (*n* = 3) were stimulated with IL-21 (20 ng/ml) and the activation of STAT3 (Tyr705) was measured by flow cytometry after 5, 10, 20, and 30 min.
(TIF)

S12 Fig **A. The STAT1 activation and B. the surface expression of LAG-3 in CD4**^**+**^ **T cells from *Ifngr***^**-/-**^ **mice following treatment with IL-21 alone, IFN-γ alone or a combined IL-21/IFN-γ treatment.** Naïve splenocytes from WT (*n* = 6) or *Ifngr*^-/-^ mice (*n* = 6) were stimulated with cytokines or left unstimulated and the activation of STAT1 (*p*Y701) was measured by flow cytometry after 10 min. The graphs are representative of two independent experiments.
(TIF)
