# Supplementary figures and images for: Interleukin 21 collaborates with interferon-γ for the optimal expression of interferon-stimulated genes and enhances protection against enteric microbial infection
Source: PLoS Pathog. 2019 Feb 28;15(2):e1007614. doi: 10.1371/journal.ppat.1007614 (PMC6413951; doi:10.1371/journal.ppat.1007614)

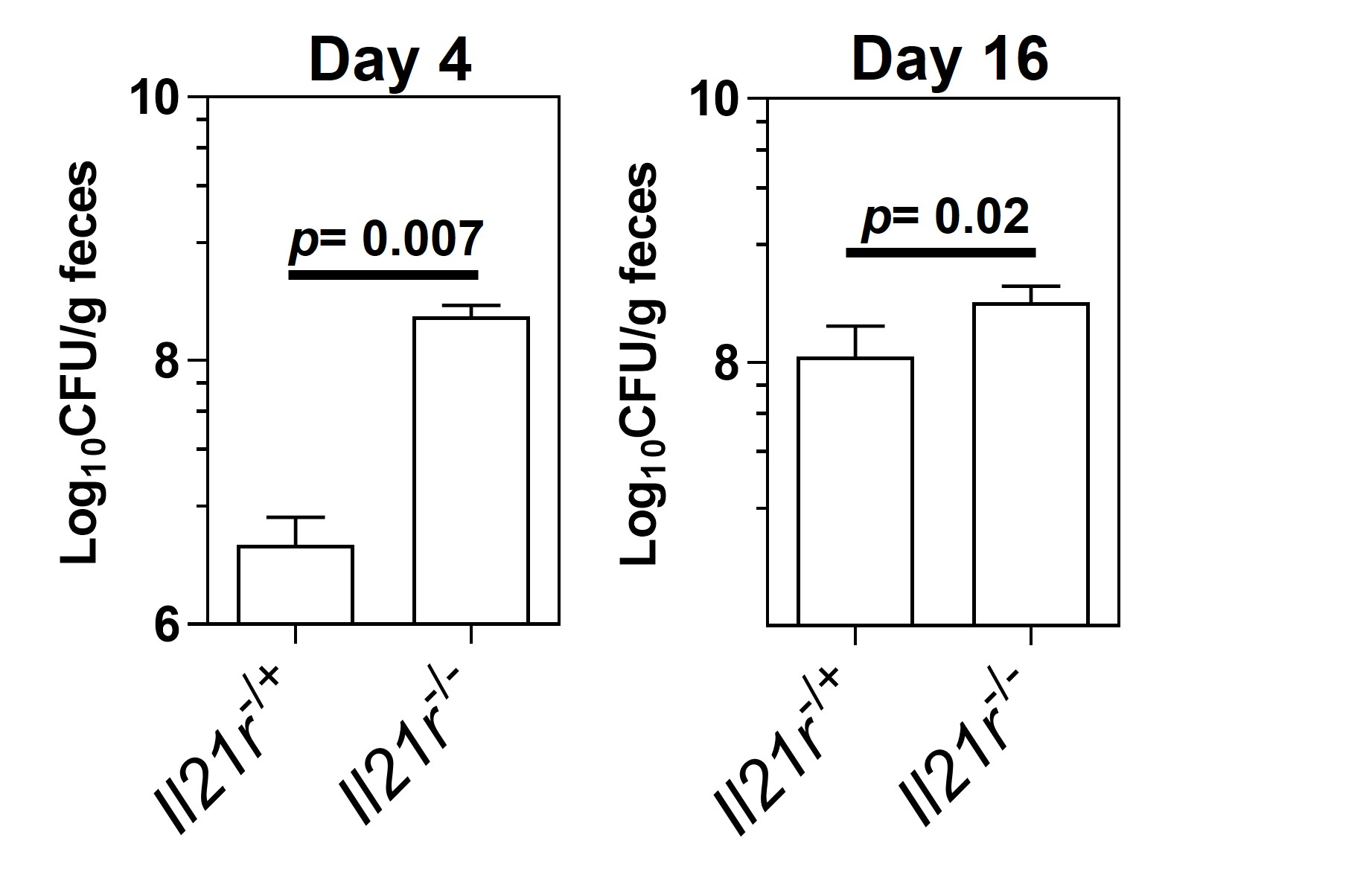

Supplement: S1 Fig — WT heterozygotes (Il21r-/-) and homozygous Il21r-/- mice were infected with C. rodentium and infection kinetics in the feces of these mice is shown as colony forming unit (CFU)/g feces. n = 5–8 mice/group. p < 0.05; p < 0.001 determined by Mann-Whitney U test. (TIF) [file ppat.1007614.s001.tif]

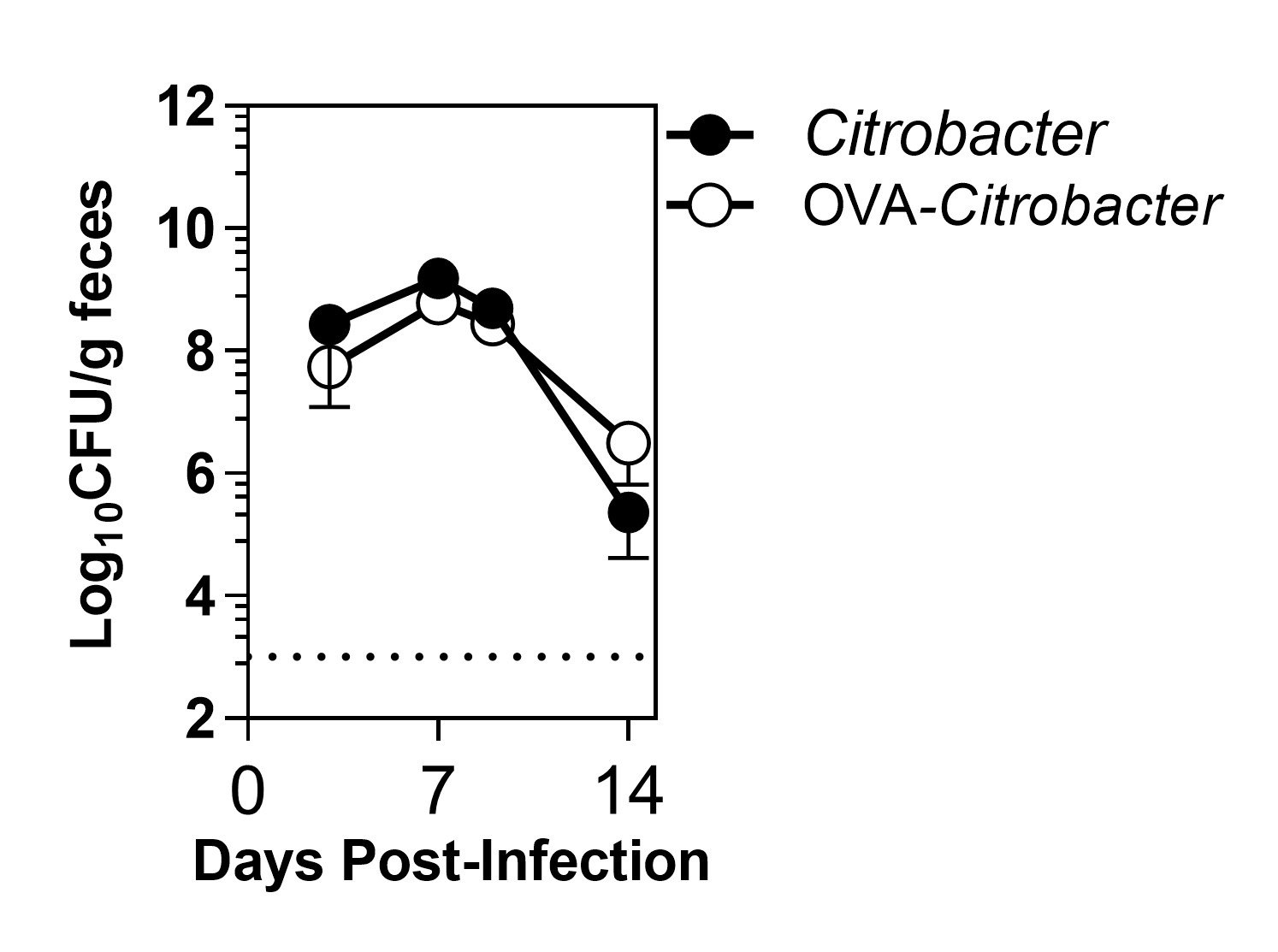

Supplement: S2 Fig — The results are the Mean ± SEM of 5 mice/group. The dashed line represents the sensitivity of the culture method. (TIF) [file ppat.1007614.s002.tif]

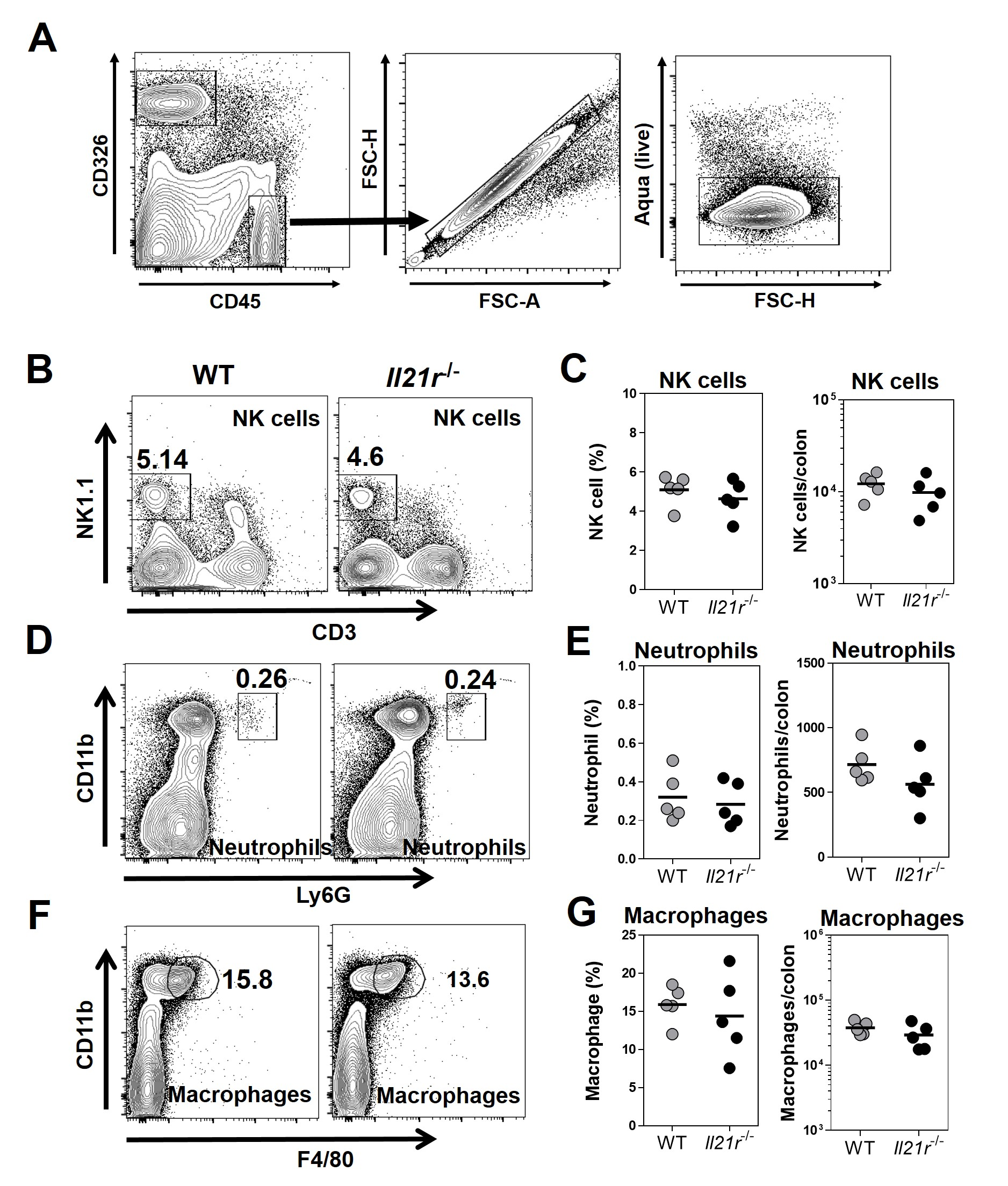

Supplement: S3 Fig — A. Gating strategy for the identification of B. natural killer (NK) cells D. neutrophils and F. macrophages in the lamina propria of the colon following infection with C. rodentium. Il21r-/- mice and WT controls had comparable percentages (left panels) and absolute numbers (right panels) of those cells in the lamina propria of the whole colon following C. rodentium infection C, E, G. (TIF) [file ppat.1007614.s003.tif]

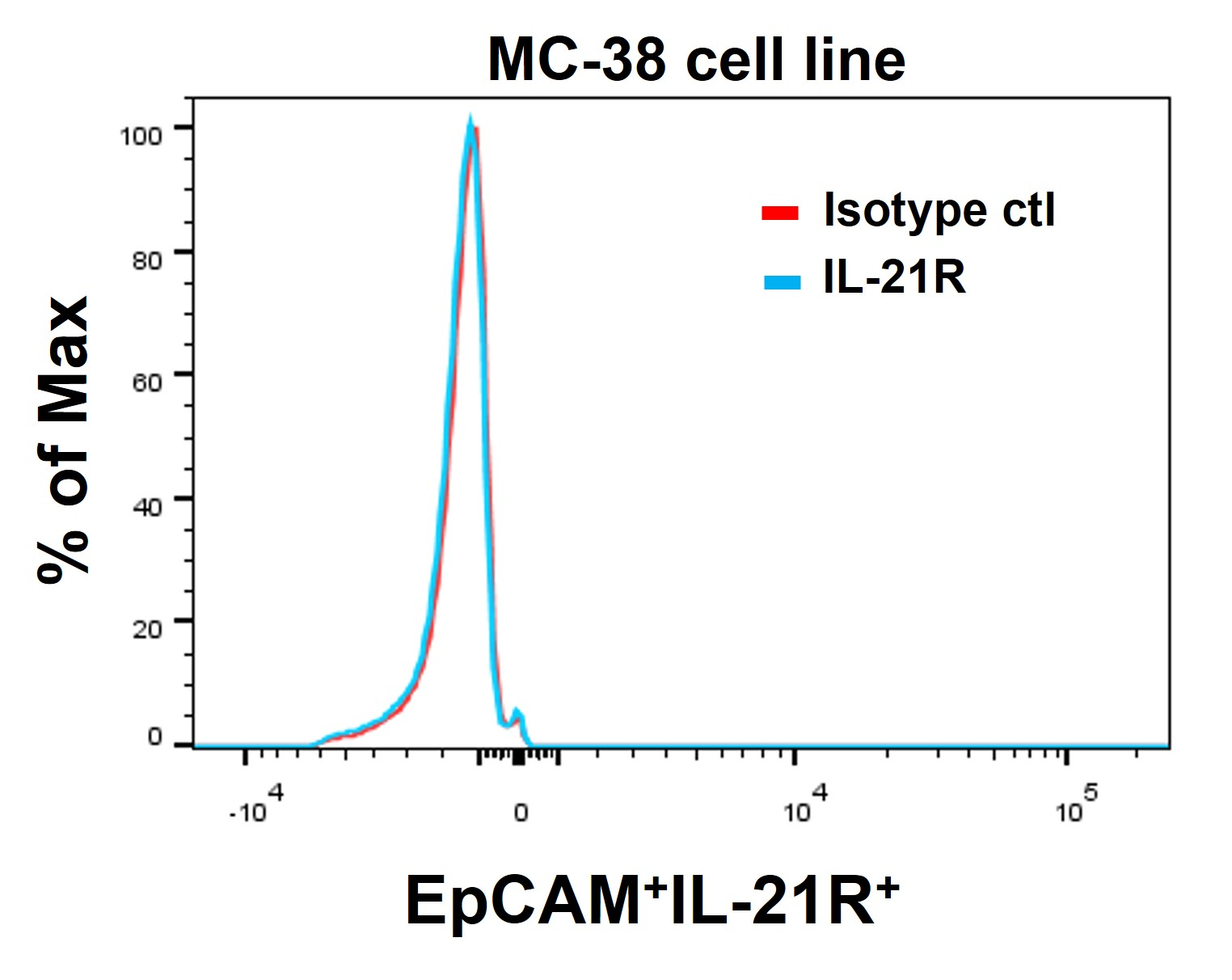

Supplement: S4 Fig — Flow cytometric analysis of surface expression of IL-21R by MC-38 carcinoma cells. (TIF) [file ppat.1007614.s004.tif]

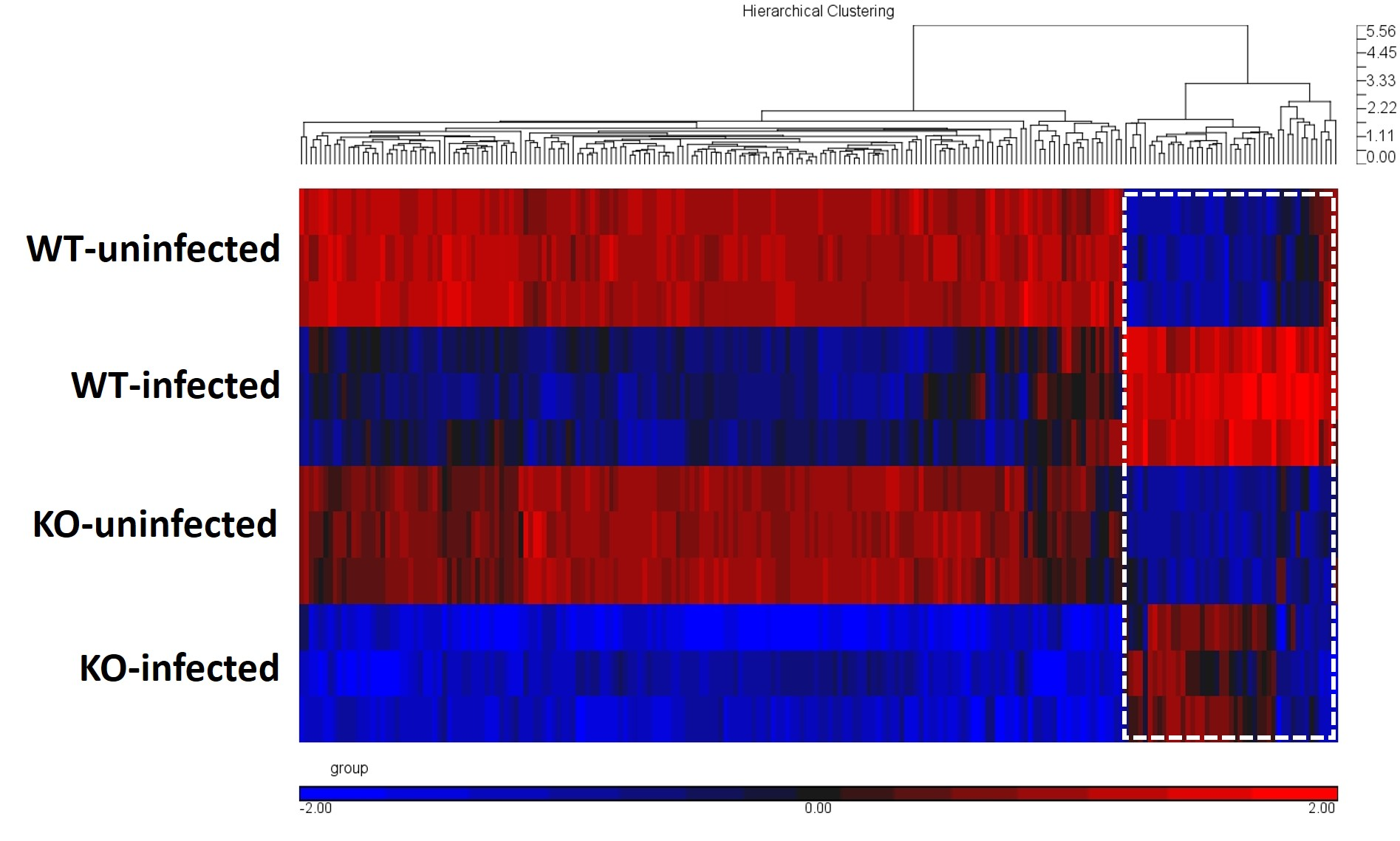

Supplement: S5 Fig — (n = 3/genotype/time-point). (TIF) [file ppat.1007614.s005.tif]

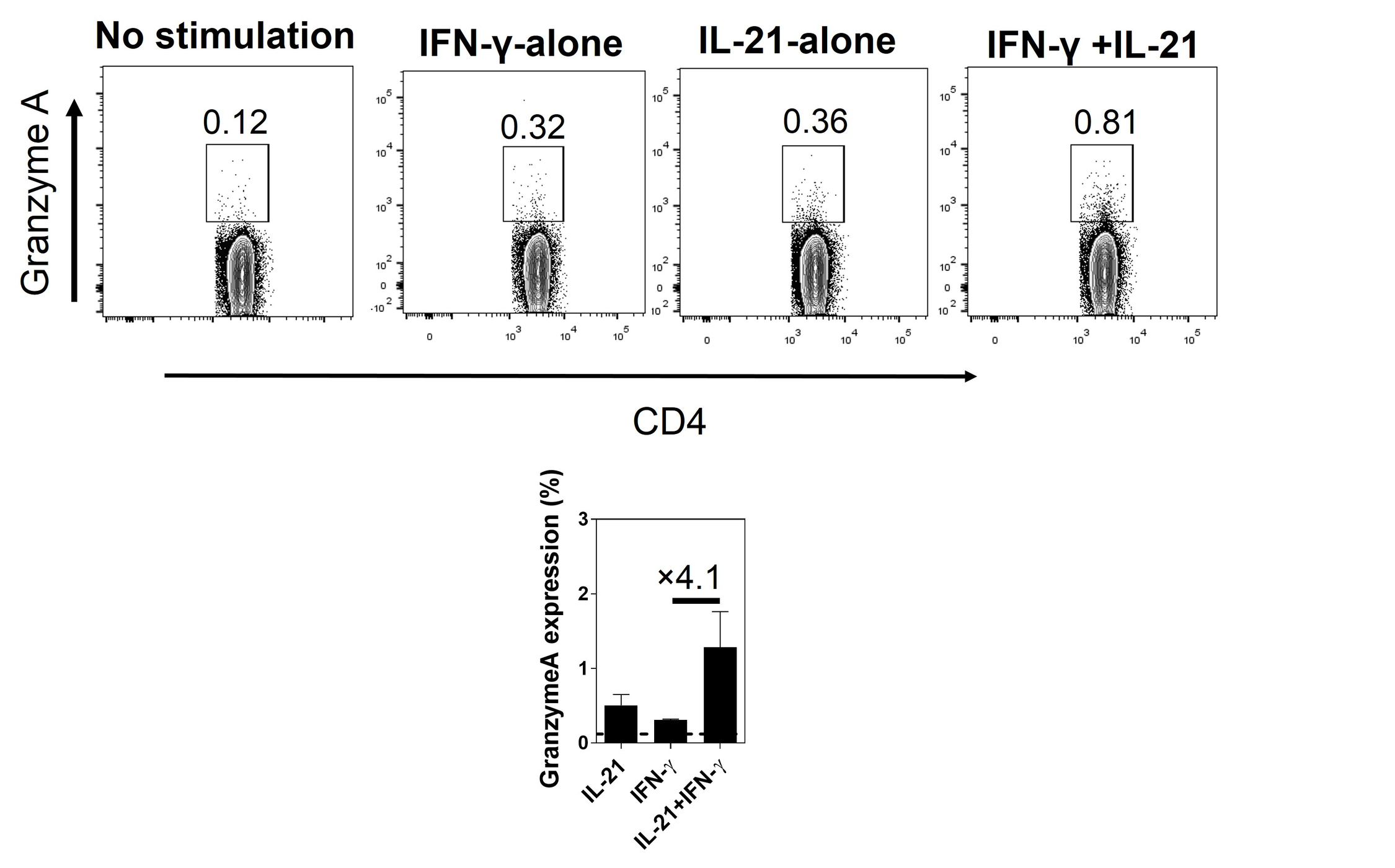

Supplement: S6 Fig — The dashed line in the graph represents the granzyme A expression in untreated splenic CD4+ T cells. (TIF) [file ppat.1007614.s006.tif]

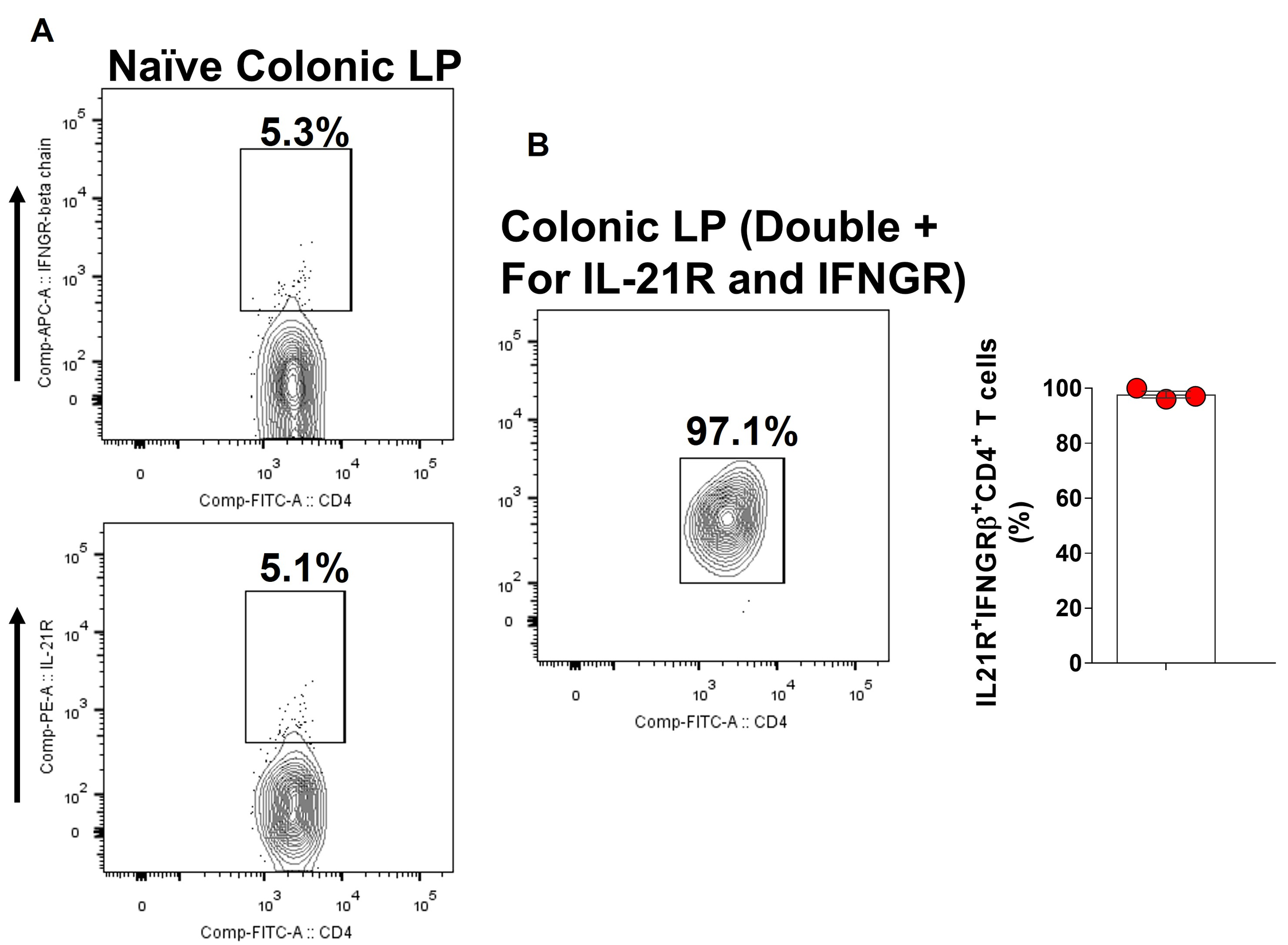

Supplement: S7 Fig — A. The flow cytometric analysis of the surface expression of IL-21R or IFN-γR by mucosal CD4+ T cells and B. CD4+ T cells positive for both IL-21R and IFN-γR enzymatically isolated from the colonic LP of naïve (uninfected) C57BL/6 mice (n = 3). (TIF) [file ppat.1007614.s007.tif]

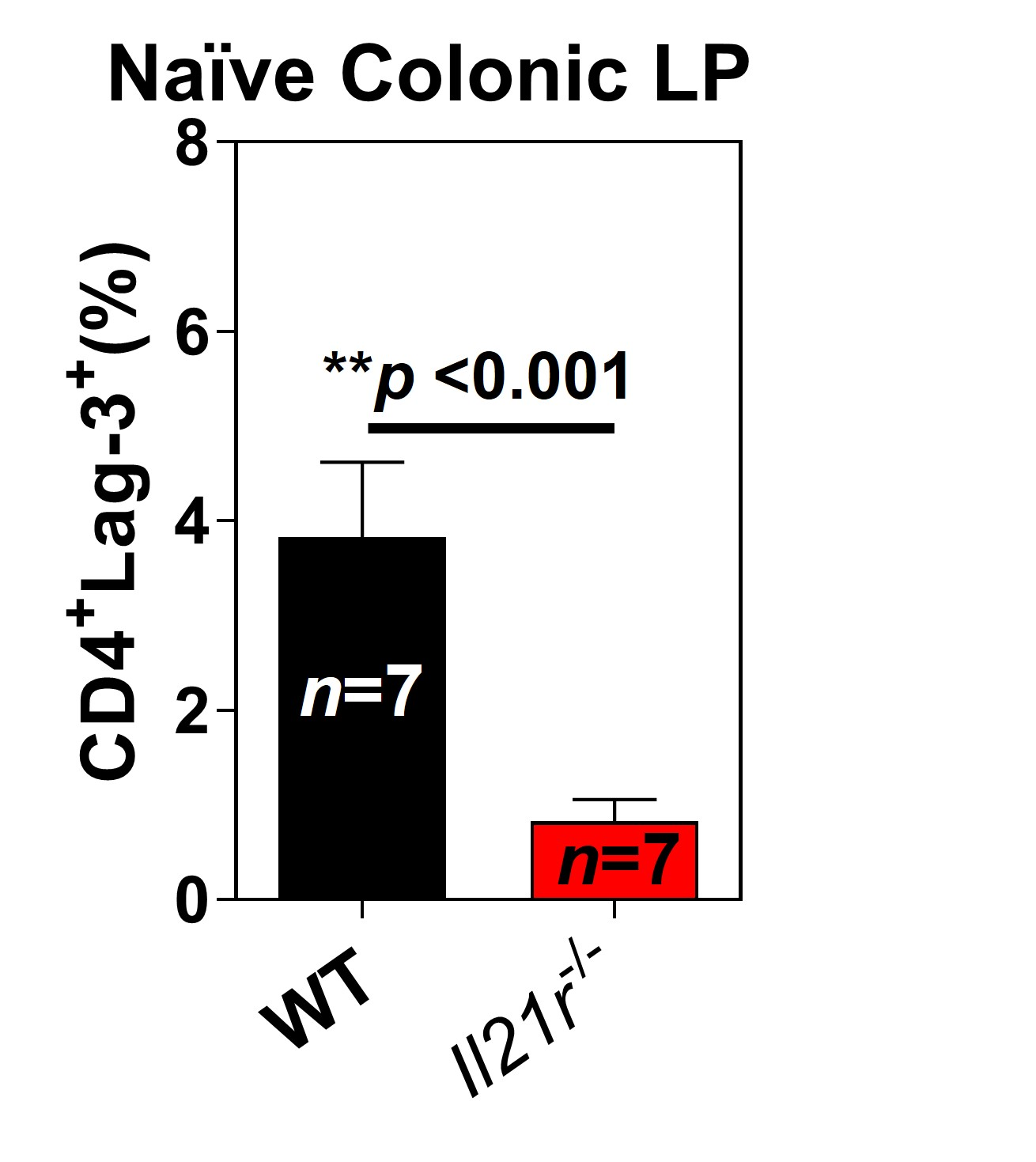

Supplement: S8 Fig — Data are the Mean ± SEM from two pooled independent experiments with a total of 7 (Il21r-/-) or 7 (WT) mice/group. **p < 0.001 determined by Mann-Whitney U test. (TIF) [file ppat.1007614.s008.tif]

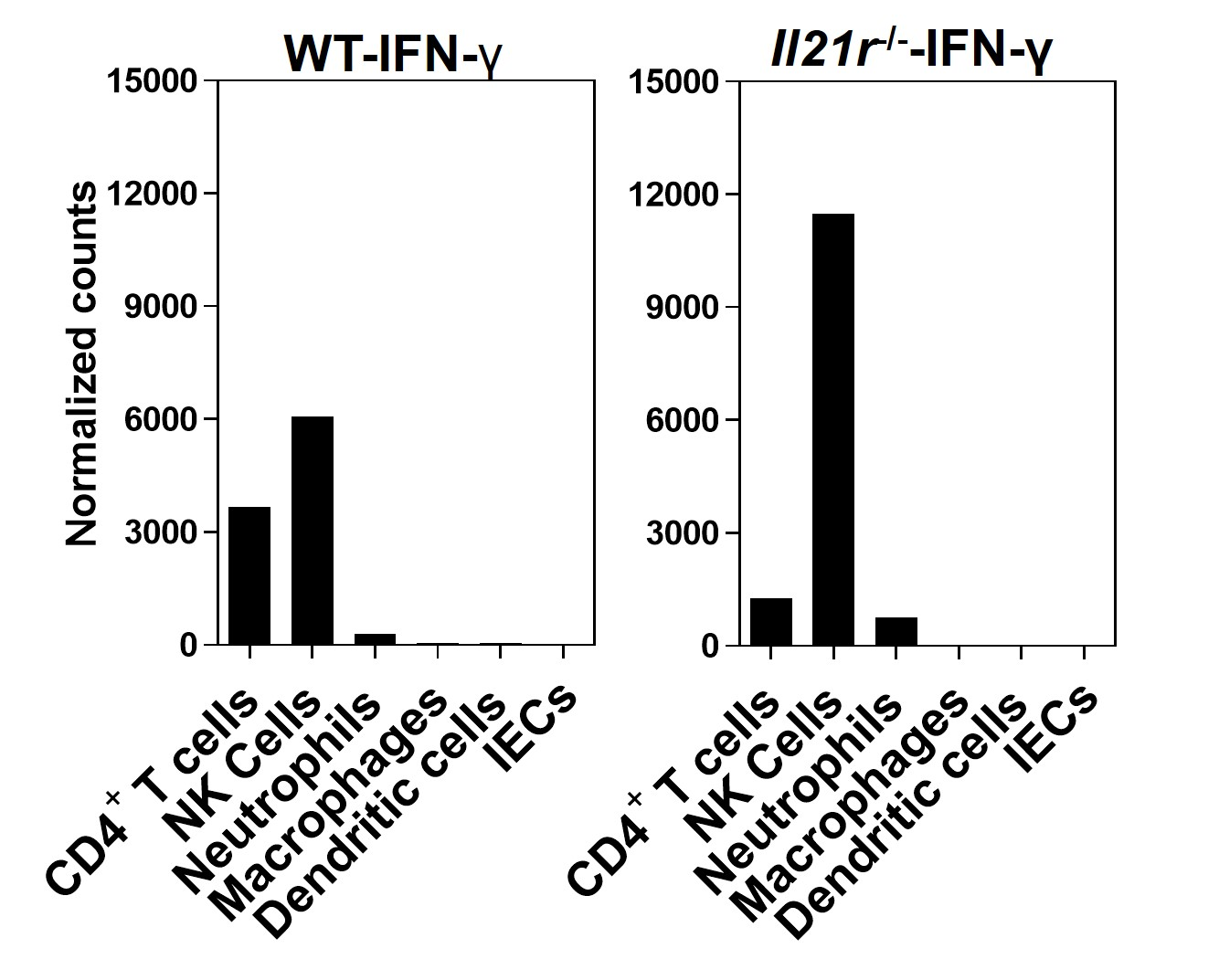

Supplement: S9 Fig — Cells were isolated enzymatically from the distal colons of mice infected 9 days p.i and were FACS-sorted. The expression of IFN-γ was measured by Nanostring at described in the Methods. (TIF) [file ppat.1007614.s009.tif]

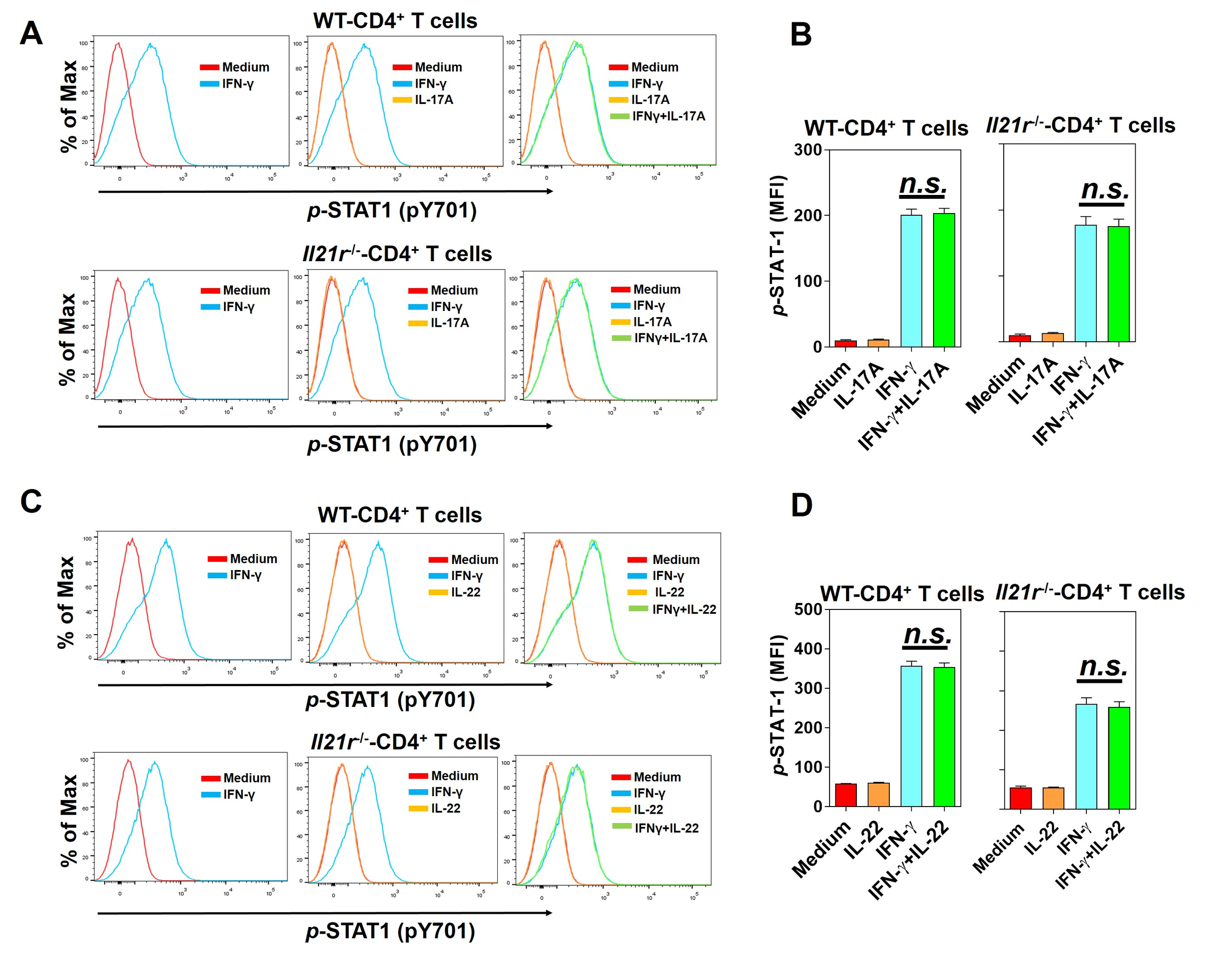

Supplement: S10 Fig — A-D. The treatment of splenocytes with a combination of IFN-γ with IL-17A or IL-22 did not result in enhanced activation of STAT1 in CD4+ T cells isolated from WT and Il21r-/- mice. The results are the Mean ± SEM of one experiment (n = 3/genotype/condition) from two independent experiments. n.s, not significant; One-way ANOVA followed by Bonferroni post-hoc adjustment test for multiple comparison. (TIF) [file ppat.1007614.s010.tif]

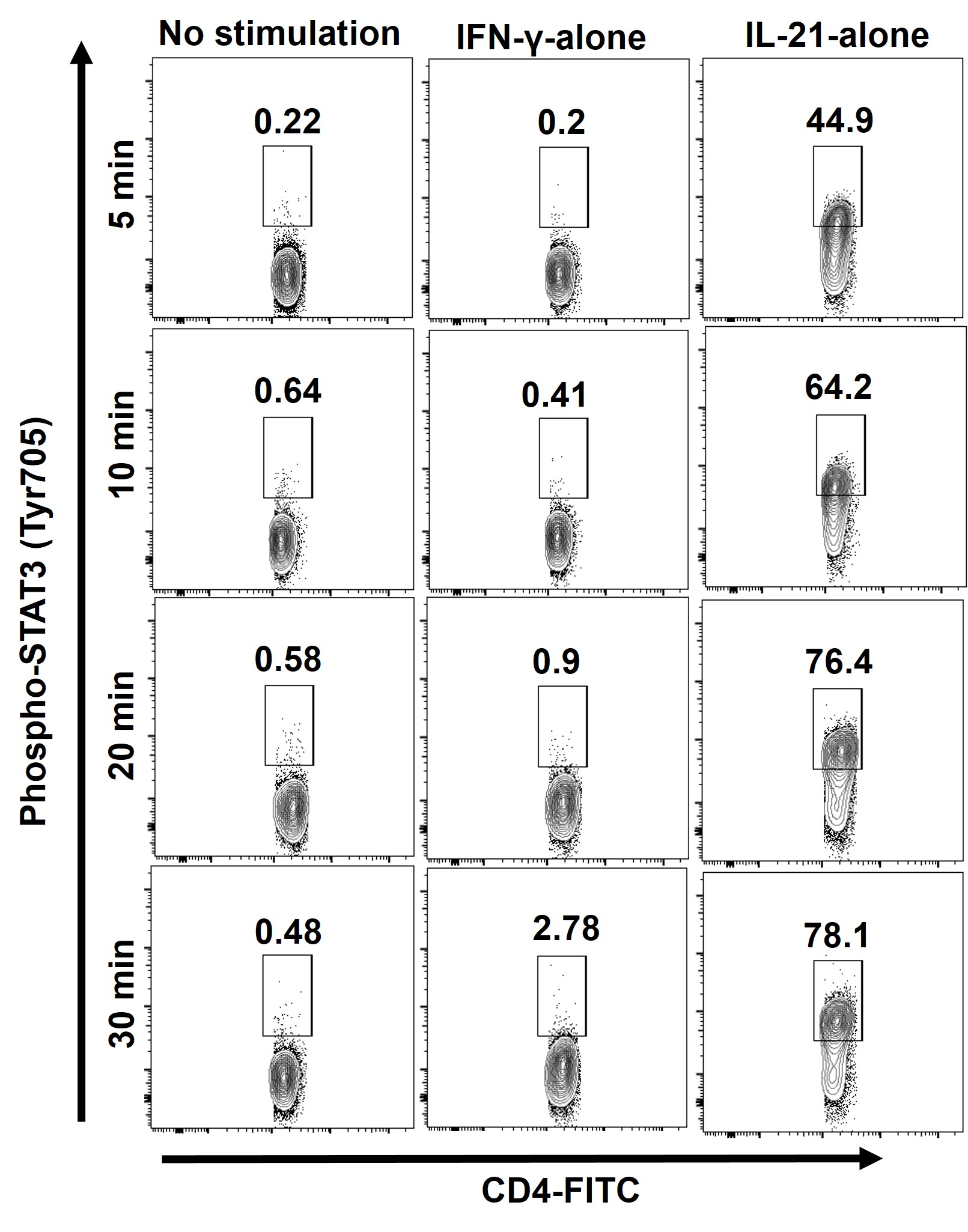

Supplement: S11 Fig — Naïve splenocytes from WT animals (n = 3) were stimulated with IL-21 (20 ng/ml) and the activation of STAT3 (Tyr705) was measured by flow cytometry after 5, 10, 20, and 30 min. (TIF) [file ppat.1007614.s011.tif]

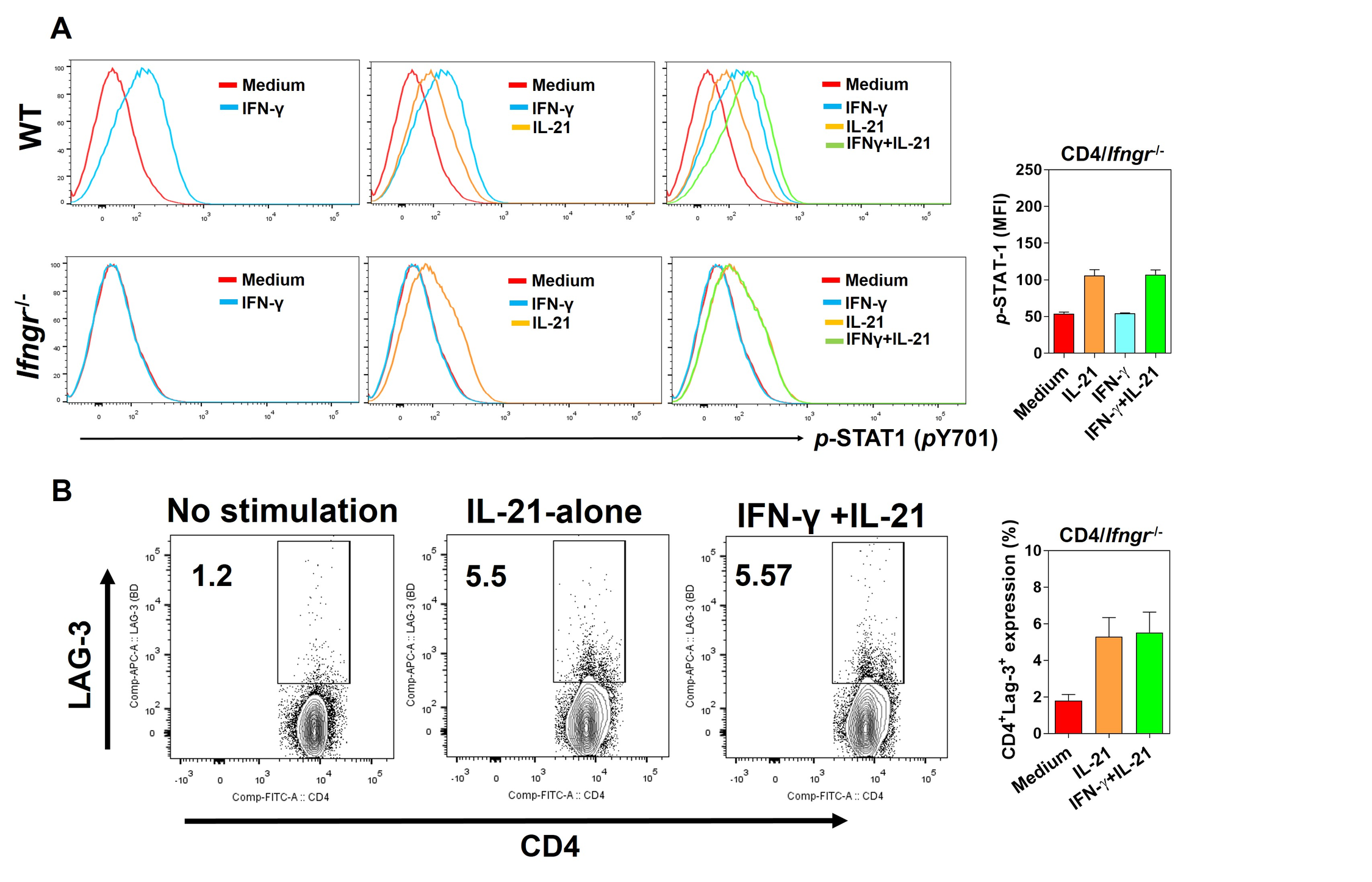

Supplement: S12 Fig — A. The STAT1 activation and B. the surface expression of LAG-3 in CD4+ T cells from Ifngr-/- mice following treatment with IL-21 alone, IFN-γ alone or a combined IL-21/IFN-γ treatment. Naïve splenocytes from WT (n = 6) or Ifngr-/- mice (n = 6) were stimulated with cytokines or left unstimulated and the activation of STAT1 (pY701) was measured by flow cytometry after 10 min. The graphs are representative of two independent experiments. (TIF) [file ppat.1007614.s012.tif]
